# 3D imaging analysis on an organoid-based platform guides personalized treatment in pancreatic ductal adenocarcinoma

**DOI:** 10.1172/JCI151604

**Published:** 2022-12-15

**Authors:** Ya’an Kang, Jenying Deng, Jianhua Ling, Xinqun Li, Yi-Ju Chiang, Eugene J. Koay, Huamin Wang, Jared K. Burks, Paul J. Chiao, Mark W. Hurd, Manoop S. Bhutani, Jeffrey H. Lee, Brian R. Weston, Anirban Maitra, Naruhiko Ikoma, Ching-Wei D. Tzeng, Jeffrey E. Lee, Ronald A. DePinho, Robert A. Wolff, Shubham Pant, Florencia McAllister, Matthew H.G. Katz, Jason B. Fleming, Michael P. Kim

**Affiliations:** 1 Department of Surgical Oncology,; 2 Department of Experimental Therapeutics,; 3 Department of Molecular and Cellular Biology,; 4 Department of Radiation Oncology,; 5 Department of Translational Molecular Pathology,; 6 Department of Leukemia,; 7 Sheikh Ahmed Center for Pancreatic Cancer Research,; 8 Department of Gastroenterology, Hepatology and Nutrition,; 9 Department of Cancer Biology,; 10 Department of GI Medical Oncology,; 11 Department of Cancer Therapeutics, and; 12 Department of Clinical Cancer Prevention, The University of Texas MD Anderson Cancer Center, Houston, Texas, USA.; 13 Department of Gastrointestinal Oncology, Moffitt Cancer Center, Tampa, Florida, USA.; 14 Department of Genetics, The University of Texas MD Anderson Cancer Center, Houston, Texas, USA.

**Keywords:** Development, Cancer

## Abstract

**BACKGROUND:**

Pancreatic ductal adenocarcinoma (PDAC) is one of the most lethal malignancies, with unpredictable responses to chemotherapy. Approaches to assay patient tumors before treatment and identify effective treatment regimens based on tumor sensitivities are lacking. We developed an organoid-based platform (OBP) to visually quantify patient-derived organoid (PDO) responses to drug treatments and associated tumor-stroma modulation for personalized PDAC therapy.

**METHODS:**

We retrospectively quantified apoptotic responses and tumor-stroma cell proportions in PDOs via 3D immunofluorescence imaging through annexin A5, α-smooth muscle actin (α-SMA), and cytokeratin 19 (CK-19) levels. Simultaneously, an ex vivo organoid drug sensitivity assay (ODSA) was used to measure responses to standard-of-care regimens. Differences between ODSA results and patient tumor responses were assessed by exact McNemar’s test.

**RESULTS:**

Immunofluorescence signals, organoid growth curves, and Ki-67 levels were measured and authenticated through the OBP for up to 14 days. ODSA drug responses were not different from patient tumor responses, as reflected by CA19-9 reductions following neoadjuvant chemotherapy (*P* = 0.99). PDOs demonstrated unique apoptotic and tumor-stroma modulation profiles (*P* < 0.0001). α-SMA/CK-19 ratio levels of more than 1.0 were associated with improved outcomes (*P* = 0.0179) and longer parental patient survival by Kaplan-Meier analysis (*P* = 0.0046).

**CONCLUSION:**

Heterogenous apoptotic drug responses and tumor-stroma modulation are present in PDOs after standard-of-care chemotherapy. Ratios of α-SMA and CK-19 levels in PDOs are associated with patient survival, and the OBP could aid in the selection of personalized therapies to improve the efficacy of systemic therapy in patients with PDAC.

**FUNDING:**

NIH/National Cancer Institute grants (K08CA218690, P01 CA117969, R50 CA243707-01A1, U54CA224065), the Skip Viragh Foundation, the Bettie Willerson Driver Cancer Research Fund, and a Cancer Center Support Grant for the Flow Cytometry and Cellular Imaging Core Facility (P30CA16672).

## Graphical abstract

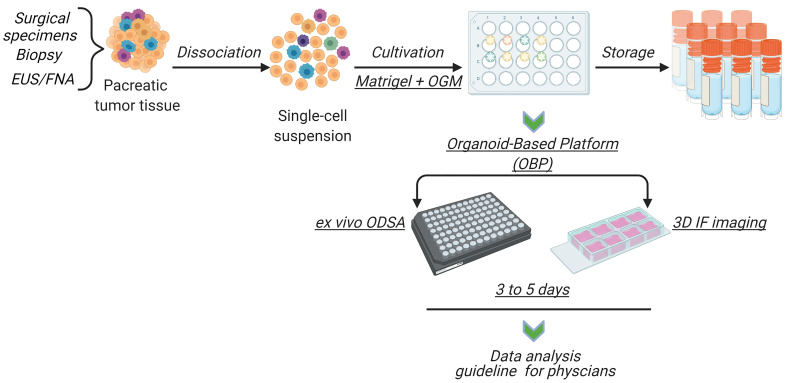

## Introduction

Pancreatic ductal adenocarcinoma (PDAC) is one of the most lethal malignancies worldwide. In the United States, researchers estimated 60,430 new PDAC cases and 48,220 PDAC-related deaths in 2021 alone. The 5-year survival rate for all stages of this cancer was reported as 10.0 % in 2021, making it the third leading cause of cancer-related deaths (1, 2). Despite modern improvements in systemic chemotherapy, the average 5-year survival rate for PDAC has remained below 10% over the past 4 decades (3). Reasons for poor PDAC outcomes include late diagnosis, early metastasis, and resistance to conventional therapeutic regimens (4, 5). Although modern chemotherapy regimens have led to improvements in patient survival (6, 7), responses to systemic therapies vary among patients, and methods to screen patient tumors for chemosensitivity prior to treatment remain limited (8). Matching patient tumors to therapies optimized for maximal efficacy could lead to improved patient tumor responses, increased rates of surgical resection, and improvements in patient outcomes.

Current model systems used to predict the efficacy of anticancer agents in PDAC have well-documented limitations. First, cultured PDAC cell lines lack interactions with other cell types in the tumor microenvironment and, therefore, may not reliably predict patient tumor responses to therapies (9). Second, although genetically engineered mouse models represent an excellent approach for studying disease progression and tumor biology, the limited repertoire of tumor mutations and lack of protracted tumor evolution may not accurately reflect human tumors known to harbor complex genetic changes that accrue over years and likely affect tumor responses to drugs (10). Finally, PDAC patient-derived xenografts (PDXs) effectively recapitulate the genetic and phenotypic alterations in human tumors but largely interact with murine stromal cells. Moreover, PDX models require significant resources to generate and maintain, with limited extrapolation to personalized medicine and real-time clinical decision-making (11–14). We previously reported ex vivo live tissue drug screening (LTSA), an ex vivo drug-screening tool that utilizes PDAC PDX tumors to conduct arrayed drug screens and to determine therapeutic efficacy with conventional drugs. However, the LTSA is largely limited to situations of relative tumor tissue abundance, as provided by surgical specimens or PDAC PDXs (15). Collectively, approaches employing cultured PDAC cells, genetically engineered mouse models, and PDX tumors have limitations that inhibit their widespread application to personalized medicine (16).

Patient-derived organoid (PDO) model systems have been used to bridge gaps between existing PDAC model systems and personalized medicine. PDOs are groups of cells assembled in 3D structures, with self-renewal and self-organization capacities, that maintain biologic characteristics similar to those of original tumors while preserving tumor genetics and heterogeneity (17–20). PDAC PDOs are generated from surgical specimens or biopsies at the time of endoscopic ultrasound and fine-needle aspiration (EUS/FNA). PDOs may therefore recapitulate a wide variety of disease stages and can be used to provide chemotherapeutic drug sensitivity profiles in individual patients, potentially matching effective chemotherapy regimens to patient tumors (19, 21). However, the predictive capacity of organoid-based drug screening has yet to be evaluated in prospective clinical trials, and more research optimizing the applications and efficacy of PDO readouts are needed. Current methods to quantify PDO responses to drug treatment include measurement of cell viability markers or markers of cellular apoptosis. Apoptosis plays an important role in the cytotoxic effect of most anticancer drugs (22, 23) and correlates with tumor volume reduction, making it a reliable surrogate marker for patient tumor response (24, 25). In previous studies, several well-established protein markers, including annexin A5, caspases, BCL-XL, and CA19-9 have been used to quantify cancer cell apoptosis (26–29). Major limitations of this approach include the acquisition of sufficient tumor tissue from patients upon which to perform drug treatment assays and the subsequent measurement of cellular apoptosis markers at the protein level. These limitations highlight the need for alternative approaches to measure cellular apoptosis, using limited tumor tissue, in translatable tumor model systems.

To better inform the selection of PDAC drug treatment regimens, we established an ex vivo organoid-based platform (OBP) to quantify antitumor efficacy and tumor-stroma cell responses in individual patients. As proof of principle of the induction of cell death pathways, we initially focused on chemo-induced apoptosis to support the accuracy and effectiveness of our OBP as a reproducible, rapid, and personalized ex vivo method of antitumor efficacy evaluation spanning 14 days. We found that image-based quantification of apoptotic markers matched colorimetric readouts of cell viability that were performed using an ex vivo organoid drug sensitivity assay (ODSA), overall permitting the facile visual assessment of drug treatment responses using very small numbers of PDOs, on average 10–20 organoids per patient. We also found that ex vivo ODSA readouts in PDOs do not significantly differ from parental patient tumor responses to neoadjuvant treatment regimens and that 3D imaging readouts also correlate with such therapeutic responses and patient survival. These results indicate that the OBP may be used to predict drug treatment responses and corresponding tumor-stroma modulation, inform clinical treatment decisions, or test therapeutic agents in a high-throughput manner.

## Results

### Construction of quantitative 3D cytoplasmic and nuclear algorithms in organoid models.

To establish an in vitro OBP, we initially developed techniques to quantify 3D structures using imaging analyses. PDOs were generated directly from patient samples and patient-derived xenograft organoids (PDXOs) using enriched media and Matrigel, as described previously (19), resulting in organoid structures containing tumor and stromal components. To identify cellular elements, organoids were stained with antibodies directed against cytokeratin 19 (CK-19) to identify tumor and ductal cells, α-smooth muscle actin (α-SMA) to identify activated fibroblasts, annexin A5 to identify cellular apoptosis, and Ki-67 to identify proliferative cells. Immunofluorescence (IF) analysis was performed on images captured with a 3D confocal microscope from approximately 10–20 unique PDXOs to develop and optimize downstream imaging protocols. Clinicopathologic features of patients from whom PDXOs were derived are summarized in Supplemental Table 1 (supplemental material available online with this article; https://doi.org/10.1172/JCI151604DS1).

**Figure 1:**
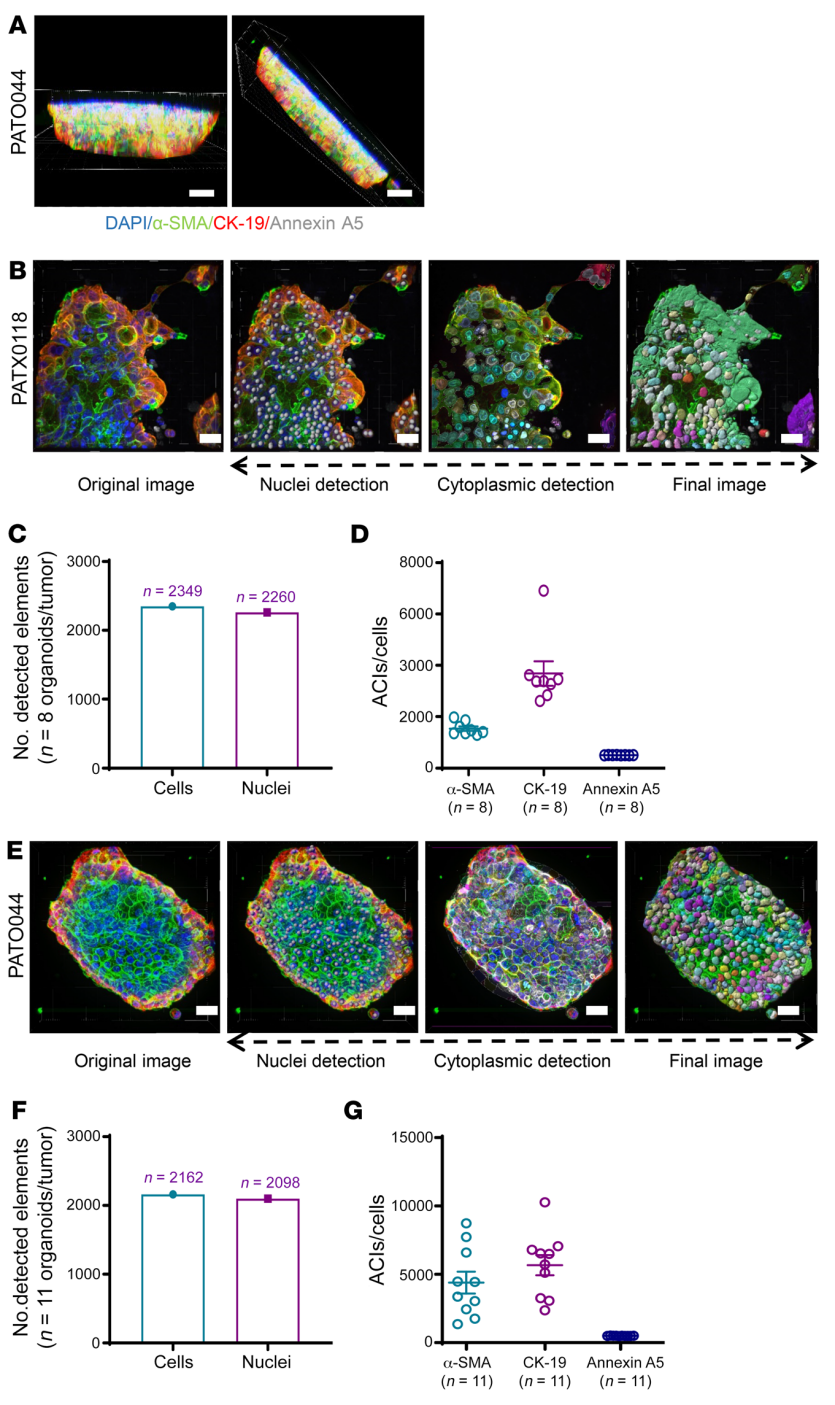
Construction and quantification of 3D cytoplasmic and nuclear algorithms in organoid models. (**A**) Thick sections (40–120 μm) of organoids were reconstructed in 3D space and segmented based on the presence of DAPI^+^ nuclei (scale bar: 20 μm). 3D reconstructed images were generated from multiple organoids per sample and compiled to generate ACIs. Details related to number of examined organoids, number of examined sections, section thickness, etc., are listed in Supplemental Table 2. (**B**) Representative images of patient-derived xenograft organoids (PDXOs) and associated nuclear and cell segmentation using cytoplasmic algorithm buildups (scale bar: 20 μm). α-SMA (green), CK-19 (red), annexin A5 (gray). (**C**) PDXO cytoplasmic imaging analysis results, showing the compiled number of detected cells (2,349) and associated nuclei (2,260) in 8 different organoids (*n* = 8) derived from PATXO118. (**D**) Measured ACIs for α-SMA, CK-19, and annexin A5 measured in 8 different organoids (*n* = 8) from PATXO118. (**E**) Representative images of patient-derived organoids (PDOs) with PATO044 and associated nuclear and cell segmentation using cytoplasmic algorithm buildups (scale bar: 20 μm). (**F** and **G**) Representative PDO imaging of cytoplasmic algorithm buildups in 11 different organoids (*n* = 11) from PATO044 and associated ACIs for α-SMA, CK-19, and annexin A5. (**D** and **G**) Data are shown as the mean ± SEM by GraphPad Prism 9.0. All 3D images were captured and merged using an Andor Revolution XDi WD Spinning Disk Confocal microscope and analyzed using Imaris software.

The total number of cells comprising tumor and stromal compartments within PDXOs and PDOs was measured after segmentation of organoids into cellular elements. Using multicolor confocal IF microscopy and Imaris software, thick sections of organoids (40–120 μm) were captured in 3D space complete with corresponding fluorescence signals (Figure 1A and Supplemental Video 1). Cell segmentation algorithms were used to quantify cellular elements on the basis of (a) the presence of a nucleus, using DAPI for nuclear segmentation and quantitation of nuclei, and (b) cytoplasmic staining, as detected by α-SMA, CK-19, and annexin A5. All organoid analyses were performed on 3D reconstructions with visual images and nuclei simultaneously identified within 40–120 μm thick organoid sections, depending on organoid size (Supplemental Table 2). This approach of simultaneous cell/nuclei segmentation and signal quantification on 3D reconstructions instead of on adjacent, individual *Z*-stack plane images precluded the examination of limited magnified fields while eliminating any “double counting” of nuclei or signal overlap between cells. Moreover, signal intensities localized at the cell membrane were not missed, as these were detected and incorporated with the cytoplasmic space; maximal signal intensities were individually measured on a per-cell basis and then averaged over many cells for average cytoplasmic intensities (ACIs). Representative PDXO imaging videos of thick 3D organoid sections are provided in Supplemental Video 1. 3D reconstructions generated from *Z*-stacks, including individual fluorophore wavelength channels and cytoplasmic algorithms, were then analyzed to quantify the expression of each protein marker using Imaris software.

Individual cells within each organoid were enumerated using cytoplasmic algorithms, and ACIs of α-SMA, CK-19, and annexin A5 were digitally quantified by PATXO118, a representative organoid (Figure 1B). The number of detected nuclei and total enumerated cells from thick organoid sections examined in 8 unique organoids (PATXO118) was 96.2% concordant (Figure 1C; nuclei, 2,349; total cells, 2,260), validating technical cell segmentation and enumeration approaches. ACIs from corresponding organoid sections (PATXO118) were detected for α-SMA, CK-19, and annexin A5 (Figure 1D). Likewise, detected nuclei and total enumerated cells analyzed in 11 organoids from PATO044 (Figure 1E) yielded excellent concordance (97.0%) (Figure 1F; nuclei, 2,162; total cells, 2,098) and detected ACIs for α-SMA, CK-19, and annexin A5 (Figure 1G).

We next sought to identify and measure cellular proliferation in organoid tumor cells when treated with cytotoxic agents. To detect cell proliferation, we used antibodies directed against Ki-67 in organoids with α-SMA and CK-19 costaining to confirm signals arising from tumor and nontumor compartments (Supplemental Figure 1A). A nuclear algorithm was generated to analyze organoids, as represented in Supplemental Video 2, shown in original PDXO imaging. Image analysis showed the number of nuclei and total cells identified for cell enumeration in 17 different organoids with 93.2% concordance (Supplemental Figure 1B; nuclei, 1,983; total cells, 2,127). In addition, nuclear imaging analysis generated the average nuclear intensity (ANI) per cell for Ki-67 (Supplemental Figure 1C; Ki-67 index shown independently) and ACIs per cell for α-SMA and CK-19 (Supplemental Figure 1D). To ensure antibody penetration into the core of examined organoids, serial images were evaluated 5 μm (1–115 μm) apart and confirmed the presence of fluorescence signals within all regions of PDOs, as represented in Supplemental Figure 2A. Supplemental Video 3 shows antibody penetration and fluorescence signals throughout the PDO. Collectively, these data indicate that cellular elements of PDAC organoids can be enumerated and that cellular markers inclusive of CK-19, α-SMA, Ki-67, and annexin A5 can be reliably detected using cytoplasmic and nuclear algorithms applied to 3D reconstructions of thick organoid sections using confocal microscopy.

### Establishment of organoid growth curves and validation of ex vivo ODSA via 3D imaging analysis.

**Figure 2:**
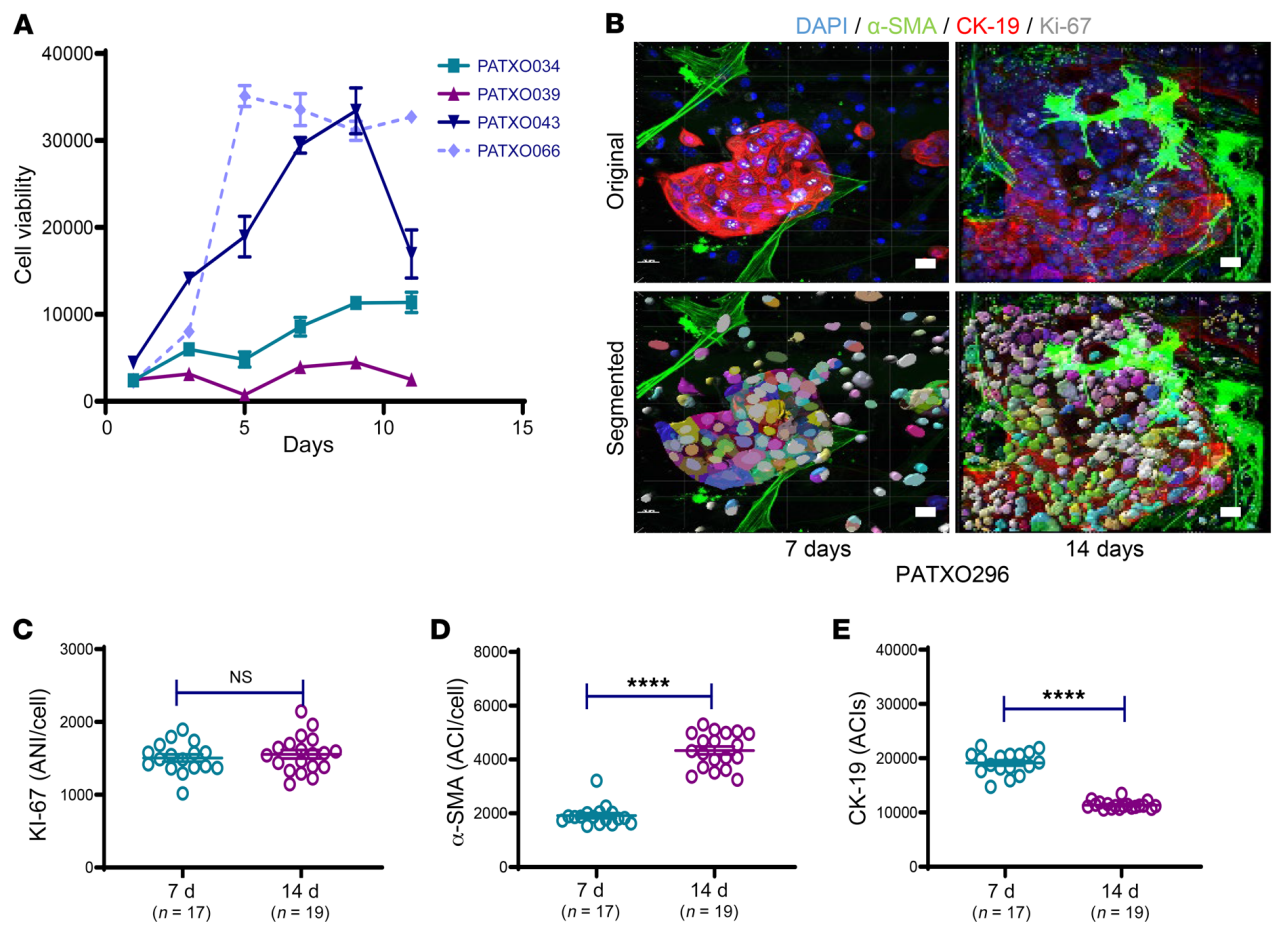
Patient-derived xenograft organoid growth curves and quantification of ACIs. (**A**) Growth curves for 4 unique patient-derived xenograft organoids (PDXOs) (*n* = 4), as measured with a cell viability assay. (**B**) A nuclear algorithm was applied to measure the average nuclear intensity (ANI) of Ki-67 7 days (left) and 14 days (right) after organoid generation. Representative original and final analysis images are shown (scale bar: 20 μm). (**C**) ANIs for Ki-67 on days 7 and 14 as measured in 18 unique organoids generated from PATXO296. (**D**) ACIs for α-SMA on days 7 and 14, as measured in 17 or 19 unique organoids generated from PATXO296. (**E**) ACIs for CK-19 on days 7 and 14, as measured in 18 unique organoids generated from PATXO296. Data are shown as the mean ± SEM by GraphPad Prism 9.0 and statistically analyzed using unpaired *t* test. ****P < 0.0001.

Using 3D imaging in PDAC organoids to identify responders to conventional chemotherapy hinges on the ability to detect and measure readouts of cell viability over drug treatment intervals. Following the establishment of rigorous 3D imaging techniques to quantity cell populations that comprise PDAC organoids, we sought to establish baseline organoid growth curves and measure the viability of cells within organoid structures over time. We initially measured PDXO growth rates and performed colorimetric readout assays for cell viability after leaving the cells in culture for 11 days (20, 30). Growth curves from 4 different PDXO models were generated over 11 days, demonstrating the heterogeneity of PDAC organoid growth (Figure 2A). To verify ongoing proliferation within an organoid, we quantified ANIs for Ki-67 in PATXO66 (Figure 2A, dashed line) on days 7 and 14 using a nuclear algorithm (Figure 2B). Although the ANIs for Ki-67 did not differ significantly after 14 days (Figure 2C), ACIs for α-SMA significantly increased (Figure 2D, *P* < 0.0001) and ACIs for CK-19 significantly decreased (Figure 2E, *P* < 0.0001). Accordingly, the ACIs of α-SMA and CK-19 over this period shifted; CK-19 became less centralized and more peripheral, with attenuated ACI, whereas the ACI for α-SMA was enhanced. To assess for similar evolutionary changes in passaged PDOs, we analyzed and compared these markers in PATO044 in passages 2, 4, and 7 (Supplemental Figure 3). Relative to passage 2, ACIs for α-SMA and annexin A5 remained stable, but CK-19 ACIs were significantly decreased in passage 7 (*P* < 0.01) due to the peripheralization of CK-19 levels in PDOs (Supplemental Figure 3, A–E), which was observed only in a small proportion of examined PDOs. The biologic significance of CK-19 peripheralization remains unknown but may signify adaptations to cell culture conditions or potential shifts in tumor cell subpopulations. To avoid such perturbations, we restricted all subsequent PDO imaging and derived data analyses to PDOs that had undergone only 2 passages and were analyzed fewer than 14 days after the most recent passage.

As we were confident that we could enumerate cell populations and measure signal intensities corresponding to a key marker of apoptosis, we next formalized an organoid-based assay workflow that spanned fewer than 14 days to screen drug efficacy ex vivo. Organoids were immediately generated upon acquisition of tumor tissue (day 0), allowed to enter a growth phase (days 0–2), and treated with standard-of-care drug regimens (days 3–5) to ultimately measure drug efficacy (days 7–14) (Supplemental Figure 4). Serial IF imaging analysis was performed within 14 days on a case-by-case basis relative to untreated controls.

**Figure 3:**
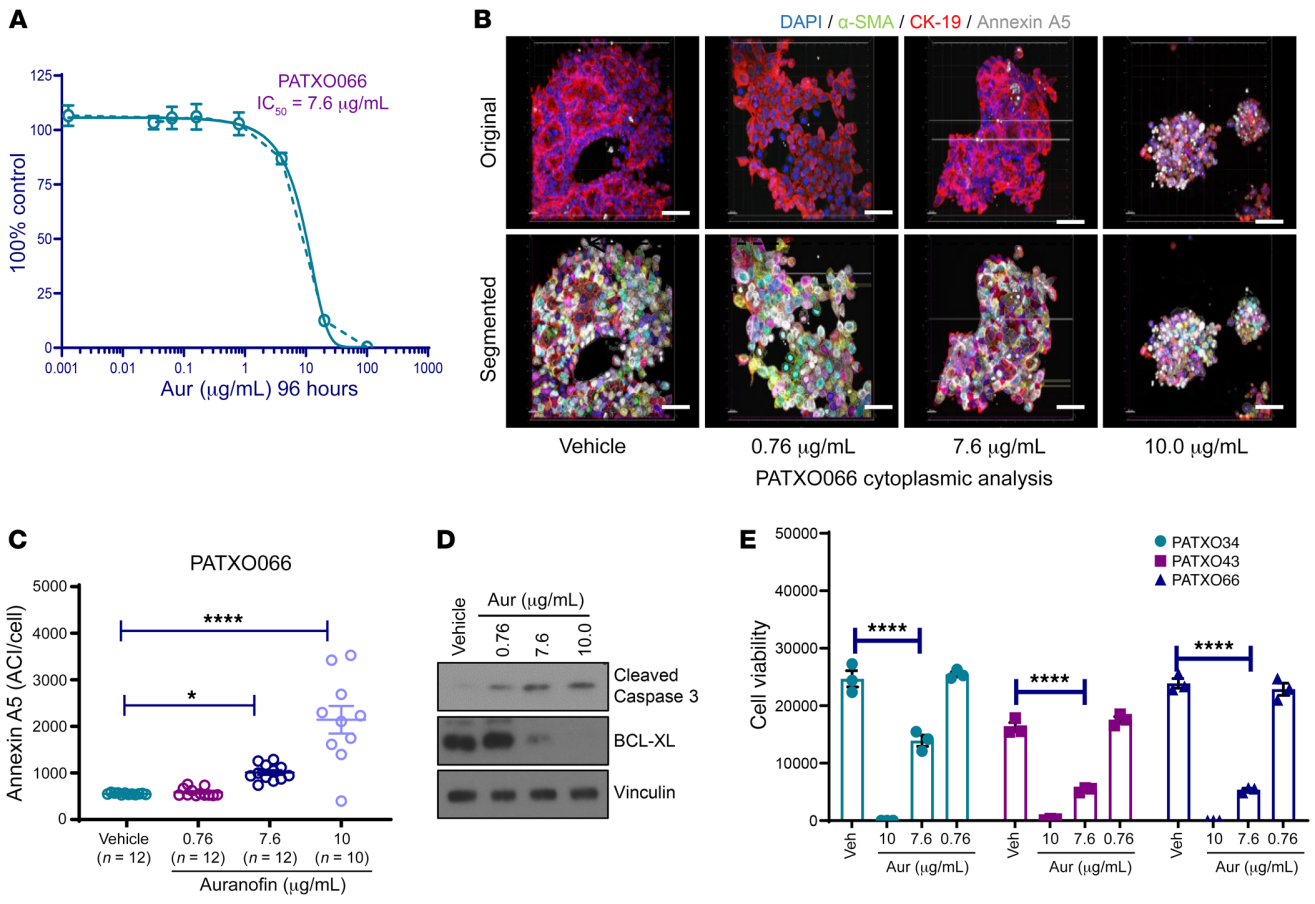
ACI quantification of annexin A5 for cell viability measurement using the ex vivo organoid drug sensitivity assay. (**A**) Auranofin (Aur) half-maximal inhibitory concentration (IC_50_; 7.6 μg/mL) in the organoid PATXO066, as measured with a cell viability assay. (**B**) Representative 3D imaging analysis of PATXO066 ACIs for annexin A5 (gray), α-SMA (green), and CK-19 (red) using the cytoplasmic algorithm after exposure to increasing doses of Aur (vehicle, 0.76, 7.60, or 10.00 μg/mL) (scale bar: 20 μm). Nuclei were counterstained with DAPI (blue). (**C**) ACIs for annexin A5 corresponding to 10–12 unique organoids (PATXO066) were measured after treatment with increasing doses of Aur (vehicle, 0.76, 7.60, or 10.00 μg/mL). (**D**) Western blot analysis showing levels of cleaved caspase-3 and B cell lymphoma–extra large (BCL-XL) as markers of apoptosis in PATXO066 after treatment with increasing concentrations of AUR (96 hours). Vinculin was used as a protein loading control. (**E**) Three different PDXOs were treated with increasing doses of Aur (vehicle, 0.76, 7.60, or 10.00 μg/mL), and cell viability was measured using the ex vivo organoid drug sensitivity assay. Data are shown as the mean ± SEM by GraphPad Prism 9.0 and statistically analyzed using ordinary 1-way ANOVA. Performed in triplicate. *P < 0.05; ****P < 0.0001.

We initially validated organoid viability following drug treatments using ex vivo ODSA colorimetric readout assays and further evaluated the matched equivalence of ODSA readouts with annexin A5 levels, as measured by 3D fluorescence microscopy. We treated representative PATX066 with auranofin (AUR), a potent gold salt used previously by our group as a positive control to induce apoptosis (15). Once the half-maximal inhibitory concentration (IC_50_) of AUR (7.6 μg/mL) in PATXO066 was calculated by colorimetric viability readouts (Figure 3A), we quantified annexin A5 expression using 3D imaging algorithms. IC_50_ values for other PATXO models, PATXO301 (3.46 μg/mL) and PATXO308 (6.88 μg/mL), were determined to fall within the same general range. Algorithm-based quantification of annexin A5 ACIs with increasing concentrations of AUR demonstrated a dose-dependent response (Figure 3, B and C). Apoptosis was further confirmed by Western blot analysis, which showed increased cleaved caspase-3 expression and reductions in the antiapoptotic protein BCL-XL, also in a dose-dependent manner (Figure 3D). The half-maximal inhibitory concentration dose of AUR was validated in 2 other PDXOs by colorimetric viability readouts using the ODSA (Figure 3E, *P* < 0.0001). Taken together, these results indicate that 3D IF imaging accurately detects and measures apoptosis in PDAC organoids, as supported by colorimetric readouts and increased apoptosis marker levels, as evaluated by Western blot.

### Quantification of chemotherapy-induced apoptosis in the ex vivo OBP.

Following OBP establishment, we used this high-throughput drug-screening platform to test the chemosensitivity of unique patient tumors based on PDAC organoid proliferation and annexin A5 levels. Given the primary objective of using the OBP to inform the selection of chemotherapy in PDAC, we focused our initial work on quantifying PDAC organoid responses to standard-of-care chemotherapy regimens. Presently, 2 FDA-approved chemotherapy regimens are primarily used to treat PDAC: (a) gemcitabine-based regimens, such as GEM plus nab-paclitaxel (GA), and (b) the combination of 5-fluorouracil, irinotecan, and oxaliplatin (FOLFIRINOX [FFX]) (6, 31). Although patients with PDAC have shown varying responses to these drug regimens, currently there are no reliable methods to predict patient responses to either regimen. Patients are often switched from one regimen to another in response to the lack of clinical responses, often reflected through failures in the reduction of carbohydrate antigen 19-9 (CA19-9) levels, a tumor marker released by pancreatic cancer cells that reflects total tumor burden and responses to neoadjuvant therapy (32). Failures in efficacy of upfront chemotherapy regimens often delays effective chemotherapy for months and may lead to interval disease progression, highlighting the need for predictive chemosensitivity assays (33). To this end, we initially sought to determine if the ODSA could render interpretable chemosensitivity data in an array of PDAC organoids derived from PDX model systems.

PDXOs were seeded into 96-well plates and matured within 3–5 days, at which point treatment with drug combinations (GEM and FFX; doses in Supplemental Tables 3 and 4) and paired imaging analyses were initiated. During ODSA establishment, we calculated GEM IC_50_ values in 5 PDXOs: PATXO053 (2.8 μM), PATXO055 (1.3 μM), PATXO066 (1.4 μM), PATXO069 (3.0 μM), and PATXO0148 (2.4 μM) (data not shown). The range of GEM treatment concentrations was based on the range of these IC_50_ values. The cytoplasmic algorithm was used to analyze the ACI for annexin A5 in PATXO66 treated with 3 GEM doses (Supplemental Figure 5A). PATXO66 cell viability decreased at all GEM doses (*P* < 0.01 at 0.16 μM, *P* < 0.0001 at 0.8 μM and 4.0 μM; Supplemental Figure 5B). In parallel, the ACI for annexin A5 increased substantially at GEM doses of 0.8 μM and 4.0 μM (*P* < 0.0001 and *P* < 0.001, respectively; Supplemental Figure 4C). Western blot analysis of treated PDXOs showed dose-dependent increases in cleaved caspase-3, especially at GEM doses of 0.8 μM and 4.0 μM, and concomitant reductions in the antiapoptosis marker, BCL-XL, after treatment with GEM (Supplemental Figure 5D). This analysis was repeated in PATXO118 with similar results (Supplemental Figure 5, E–H). Collectively, these data indicate that responses to drug treatment can be reliably captured using 3D IF imaging and that resulting data are concordant with conventional plate reader viability assays.

We next applied the ex vivo ODSA to evaluate PDXO responses to GEM and 5-fluorouracil–based drug regimens. PATXO066 and PATXO118 were treated with combinations of GEM/paclitaxel (GEM/PAC) and GEM/cisplatin, which showed increased responses relative to treatment with vehicle (Supplemental Figure 6, A and B, *P* < 0.0001). PDXO responses were tested in PATXO053 and PATXO060; treatment with FFX resulted in marked reductions in cell viability (Supplemental Figure 6C, *P* < 0.01 or *P* < 0.0001). Relative resistance to GEM/PAC and FFX was detected in PDOs generated from a patient FNA (FNA34), demonstrating response heterogeneity between PDAC organoids tested in the ODSA (Supplemental Figure 6D). Confident that 3D IF imaging results were concordant with ODSA results and that responder and nonresponder PDAC organoids were identified in the ODSA, we next sought to apply this technology to organoids derived from a larger cohort of patient tumors. PDOs were generated from tumors acquired at the time of surgical resection or from core biopsies acquired at the time of EUS. PDO clinicopathologic features of patients with PDAC from whom PDOs were derived are summarized in Supplemental Table 5.

**Figure 4:**
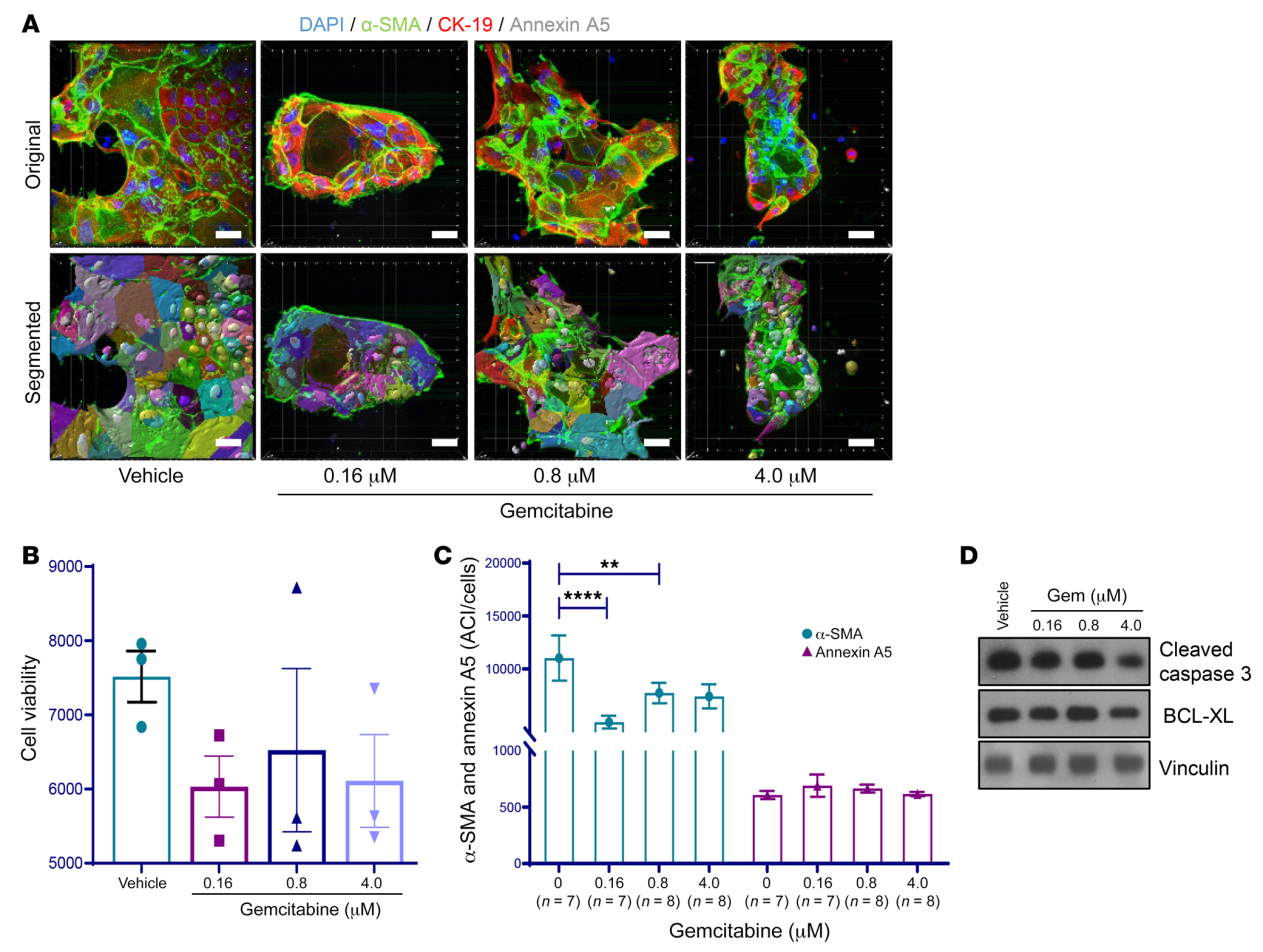
3D immunofluorescence imaging analysis and quantification of PATO020 after exposure to gemcitabine. (**A**) Representative images of PATO020 following treatment with increasing concentrations of gemcitabine (Gem) or vehicle (scale bar: 20 μm). Nuclei were counterstained with DAPI (blue). (**B**) Ex vivo ODSA results for PATO020 indicating pan-resistance to GEM treatment. Performed in triplicate. (**C**) ACIs for α-SMA and annexin a5 in 7–8 different PDOs from PATO020. The ACI for α-SMA decreased after treatment with 0.16 μM and 0.8 μM Gem compared with vehicle control. The ACI for annexin A5 did not change after Gem treatment, indicating pan-resistance. (**D**) Western blot analysis of apoptosis markers cleaved caspase-3 or B cell lymphoma–extra large (BCL-XL) in PATO020 after treatment with increasing concentrations of Gem (96 hours). Data are shown as the mean ± SEM by GraphPad Prism 9.0 and statistically analyzed using ordinary 1-way ANOVA. **P < 0.01; ****P < 0.0001.

**Figure 5:**
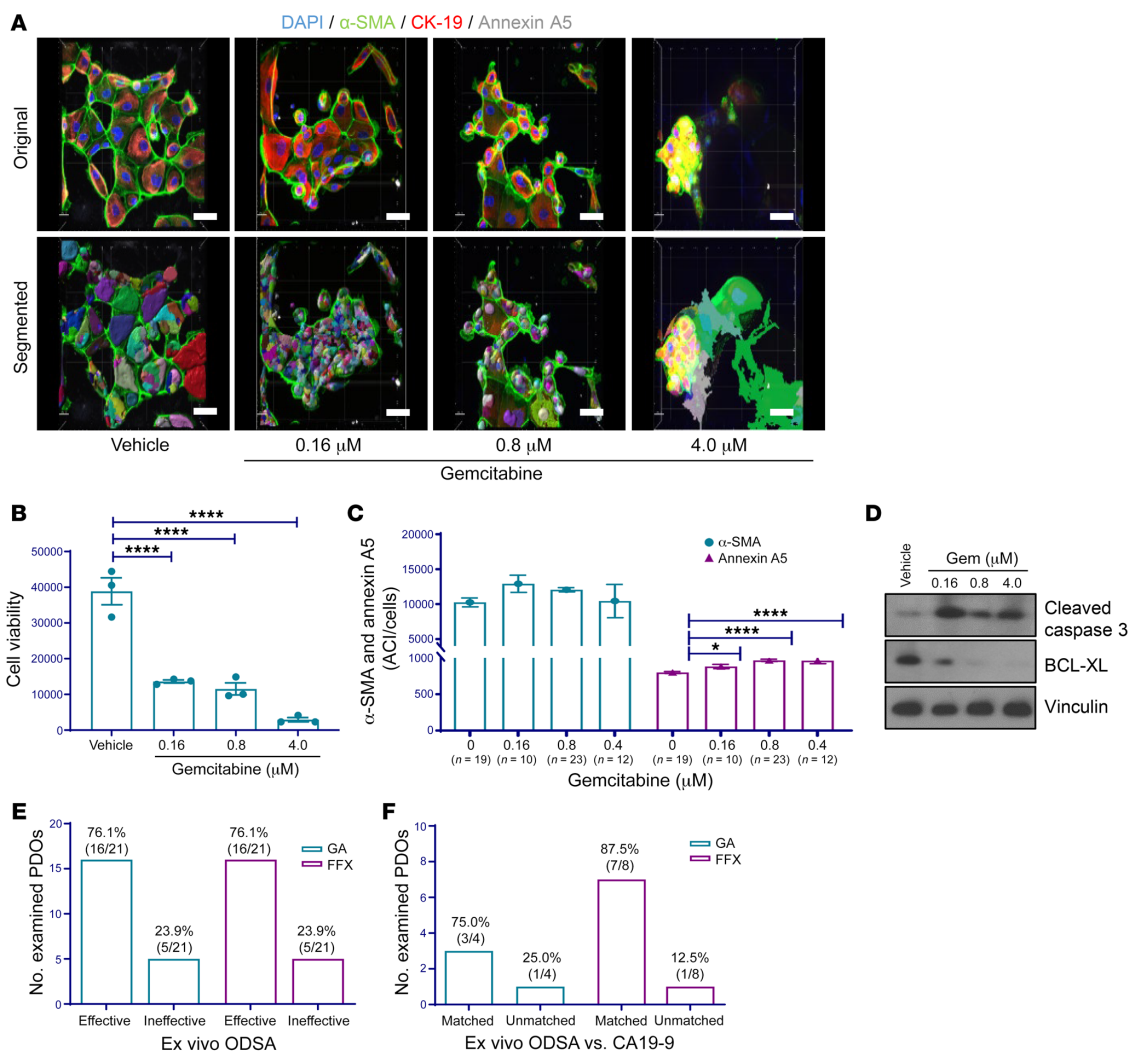
3D immunofluorescence imaging analysis and quantification of PATO015 after exposure to gemcitabine. (**A**) Representative images of PATO015 following treatment with increasing concentrations of gemcitabine (Gem) or vehicle (scale bar: 20 μm). Nuclei were counterstained with DAPI (blue). (**B**) Ex vivo ODSA results for PATO015 indicating pan-sensitivity to GEM treatment. Performed in triplicate. (**C**) ACIs for α-SMA and annexin A5 in 10–23 different PDOs from PATO015 after treatment with increasing concentrations of Gem. Compared with vehicle, the ACI for annexin A5 increased relative to control after Gem treatment, indicating sensitivity. (**D**) Western blot analysis of apoptosis markers cleaved caspase-3 or BCL-XL in PATO015 after treatment with increasing concentrations of Gem (96 hours). (**E**) Summary of PDO sensitivity to PDAC treatment regimens gemcitabine/paclitaxel (GA) or FOLFIRINOX (FFX), as measured by the ex vivo ODSA at increasing drug doses (see full data in Supplemental Figure 6, E and F). (**F**) Consistency of ex vivo ODSA-determined effectiveness with carbohydrate antigen 19-9 (CA19-9) status (“matched” indicates consistent findings). Data are shown as the mean ± SEM by GraphPad Prism 9.0 and statistically analyzed through ordinary 1-way ANOVA. *P < 0.05; ****P < 0.0001.

An optimized cytoplasmic algorithm was created to analyze and quantify ACIs in PDOs. The number of PDOs examined by 3D IF microscopy varied due to model-specific differences in PDO size and abundance in 3D culture (details listed in Supplemental Table 2). As a representative nonresponder model, PATO020 was treated with 3 GEM doses or a vehicle control for 4 days and then analyzed using 3D imaging quantification based on 7–8 unique PDOs for each condition (Figure 4A and Supplemental Table 2). Following treatment with increasing doses of GEM, PDOs showed no reductions in viability in the ex vivo ODSA (Figure 4B). Concurrently, 3D imaging analysis showed no differences in annexin A5 ACIs but did show reductions in α-SMA ACIs following GEM treatment, indicating ongoing treatment effects (Figure 4C). Accordingly, Western blot analysis showed no significant changes in apoptotic marker levels (Figure 4D). ODSA analyses of PATO015, a representative responder model based on 10–23 unique PDOs per treatment group, exhibited significant reductions at all 3 GEM doses (Figure 5, A and B, *P* < 0.0001). Likewise, 3D IF analysis demonstrated significant increases in annexin A5 ACIs and stable α-SMA ACIs with increasing doses of GEM treatment (Figure 5C). Western blot analysis confirmed increased levels of cleaved caspase-3 and concomitant reductions in BCL-XL levels after treatment (Figure 5D). The significance of stable α-SMA ACIs during drug treatments remains unknown but might be due to temporal differences in annexin A5 expression relative to tumor cells. Alternatively, because PDAC stroma is supportive of tumor cell growth and a well-described modulator of therapy resistance, stable α-SMA levels in CAFs during drug treatment may itself be a marker of treatment resistance and requires additional validation in larger patient cohorts

Collectively, our results show that 3D imaging of PDOs is a complementary technique to the ODSA to measure responses to drug treatments and requires only 10–20 unique PDOs in which to accurately quantify annexin A5 levels as a marker of drug-induced apoptosis. Such a strategy is particularly useful when only small amounts of tumor tissue are available to generate PDAC organoids, as is the case in our experience for most EUS/FNA and resected PDAC cases. Accordingly, our OBP (ODSA and/or 3D IF) may be used as an independent platform to quantify and analyze antitumor efficacy and tumor-stroma cell responses in individual PDAC tumors (30).

### Ex vivo ODSA results compared with neoadjuvant treatment regimen effectiveness measured by CA19-9 status.

To initially determine relative sensitivities to modern chemotherapy regimens, we treated 21 PDOs (19 resected tumors and 2 EUS/FNA specimens) with GEM/PAC or FFX and measured PDO responses using the ex vivo ODSA. Doses for the ODSA were reduced from 4.0 μM to 0.5 μM to diminish the toxicity of combined agents (Supplemental Tables 3 and 4). Static viability reduction thresholds to define responders or nonresponders to drug treatments are hotly debated in the field and currently remain without consensus or strong statistical justifications. To address this field gap and to mitigate subjectivity when assigning responder or nonresponder status in our work, we generated an initial treatment matrix to measure treatment responses: (a) over a spectrum of treatment doses (low, medium, high; Supplemental Tables 3 and 4); (b) across a spectrum of 21 different PDO models; and (c) across a spectrum of viability-reduction thresholds (10%, 20%, 30%, 40%) that denote drug treatment responses (Supplemental Figure 6E). This system was designed to provide depth and granularity to PDO responses following treatment with two standard-of-care treatment regimens, GEM/PAC or FFX, while allowing us to objectively observe natural clusters of responder or nonresponder PDO models as a function of treatment dose. Based on such clusters of responders or nonresponders, we established unbiased percentage of viability reduction thresholds, indicative of objective PDO treatment responses, that were subsequently validated by patient tumor responses.

Responder, intermediate, and nonresponder statuses were assigned based on a binary system in which 1 equaled response and 0 equaled no response to treatment with increasing doses of drugs (Supplemental Figure 6E). Most importantly, all PDO models from patients who demonstrated clinical responses through CA19-9 reductions (PDOs in bold) naturally grouped as responders (red) or intermediate (yellow) responders to GEM/PAC or FFX treatment, validating this initial approach. At 10% and 20% viability reduction thresholds — in which PDOs were deemed responders by more than 10% or more than 20% reductions in cell viability, respectively — PDO treatment with GEM/PAC resulted in responses skewed toward responders and intermediate responders; the only PDXO nonresponsive to treatment was PATO061 (Supplemental Figure 6E, top). More stringent responder thresholds of 30% and 40% resulted in a better balance of responder/intermediate/nonresponder models, with a distinct, observable inflection of intermediate to nonresponders at the transition from 20% to 30% thresholds (Supplemental Figure 6E, top, yellow to green). Based on a more balanced arrangement of responder/intermediate/nonresponder readouts and the objective presence of an inflection point between intermediate and nonresponder PDOs above 20%, we interpreted a 30% threshold in the GEM/PAC group to represent an objective response threshold. Similarly, when analyzing PDOs treated with FFX, we noticed objective response inflections in proportions of responder/intermediate responder models when transitioning from 30% to 40% and from 10% to 20% thresholds (Supplemental Figure 6E, bottom). As such, 40% and 10% thresholds in the FFX treatment matrix represented outliers, with skewed readouts toward resistant and responder models, respectively. We therefore interpreted 20% or 30% viability reduction thresholds in the FFX group, representing a distinct cluster of balanced responder/intermediate/nonresponder models bracketed by obvious response inflections, to represent objective response thresholds. Because overlap of objective response thresholds between GEM/PAC and FFX data sets occurred only with a 30% viability reduction threshold, and PDO responses using a 30% viability reduction threshold universally correlated with patient tumor responses, this threshold was tentatively identified as optimal for the ODSA.

We next sought to distill our responder or nonresponder matrix into a binary, responder/nonresponder system to simplify readout interpretation and validate the optimal percentage viability reduction threshold of 30%. We converted all intermediate responders to responders and again assessed for naturally-occurring clusters of responder or nonresponder model among tested PDOs (Supplemental Figure 6F). Following GEM/PAC treatment, an objective inflection in nonresponder to responder status was again observed when transitioning from 20% to 30% thresholds (Supplemental Figure 6F, top), indicating represents 30% an objective response threshold. No distinct transitions between responder and nonresponder models were observed after FFX treatment. However, in the FFX treatment group, a 30%, but not 40%, threshold was sufficient to match PATO082 as a responder in our assay system with patient tumor response (Supplemental Figure 6F, bottom). Taken together, orthogonal analysis of our data sets objectively indicated 30% as an optimal threshold to discern responder from nonresponder PDOs following GEM/PAC or FFX treatment that best matched patient tumor responses. Therefore, a 30% viability reduction was ultimately selected as the threshold between responder and nonresponder ODSA readouts (Supplemental Figure 7, A and B).

Table 1 shows ODSA-determined categorical responses, patient neoadjuvant treatment regimens, and CA19-9 status before and after neoadjuvant treatment and at last follow-up. GEM/PAC or FFX treatments were considered effective if a 30% viability reduction was observed in any of the 3 treatment doses in the ex vivo ODSA or in 16 of 21 PDOs (76.1%) (Figure 5E). We then compared the ODSA-determined effectiveness of GEM/PAC or FFX with the effectiveness of GA or FFX neoadjuvant treatment regimens in patients with PDAC, as determined by reductions in serum CA19-9 levels (34). This analysis excluded 5 PDOs from patients with normal-range CA19-9 (PATO032, PATO035, PATO077, PATO062, and PATO066) and 5 PDOs from patients who did not receive neoadjuvant therapy (PATO081, PATO083, PATO084, FNA26, and FNA27), as shown in boldface in Table 1. ODSA results showed that GEM/PAC was effective in 3 of 4 PDOs in which GA showed clinical effects (75 %), reflected by decreased CA19-9 levels (treatment response, PATO073, PATO044, PATO080, and PATO075). ODSA results also showed that FFX was effective in 7 of 8 PDOs in which FFX resulted in clinical responses (87.5 %), as reflected by reductions in CA19-9 levels (treatment response, PATO068, PATO043, PATO069, PATO082, PATO080, PATO075, PATO061, and PATO071; Figure 5F). Tumor response rates observed in patients following treatment with chemotherapy (GA or FFX) demonstrated no statistically significant differences from responses observed in derived PDOs after treatment with identical drug regimens in the ODSA, as calculated by exact McNemar’s test (*P* = 0.99). Taken together, ex vivo ODSA results reflected the effects of neoadjuvant treatment regimens in most patients and might provide potential strategic adjuvant treatment guidelines for clinicians.

Follow-up CA19-9 levels increased in the patients from whom PDOs PATO044 and PATO080 were derived, indicating that adjustment of clinical adjuvant regimens could be considered in these patients. For example, CA19-9 levels increased in a patient treated with MK/capecitabine (PATO072), indicating a lack of efficacy, while the ODSA indicated sensitivity to GEM/PAC and FFX. Such data could be interpreted by clinicians as a potential indication to switch treatment regimens. For 3 treatment-naive patients, the ODSA showed substantial effectiveness of GEM/PAC in PATO081, FFX in PATO083, and both GEM/PAC and FFX in PATO084. Similarly, for 2 EUS/FNA PDOs, the patients’ CA19-9 levels dramatically decreased after adjuvant FFX (FNA26) and GA (FNA27) regimens, matching the ODSA sensitivity results (Table 1). These results again indicate that the ODSA could potentially inform the selection of GA or FFX regimens for adjuvant treatment in individual patients with PDAC.

These data demonstrate heterogeneous responses to GA and FFX therapy vis-à-vis ODSA readouts that correlate with treatment responses in patients. Moreover, the observation that the ex vivo ODSA can agnostically stratify PDOs into groups that demonstrate preferential, dual, or a lack of sensitivity to the GA and/or FFX treatment regimens indicates a potential role for the ODSA in the identification of efficacious chemotherapy regimens in the neoadjuvant or adjuvant treatment of PDAC.

### PDOs established from EUS/FNA PDAC specimens and tumor-stroma cell ratios in PDOs from patients who received neoadjuvant therapy.

**Figure 6:**
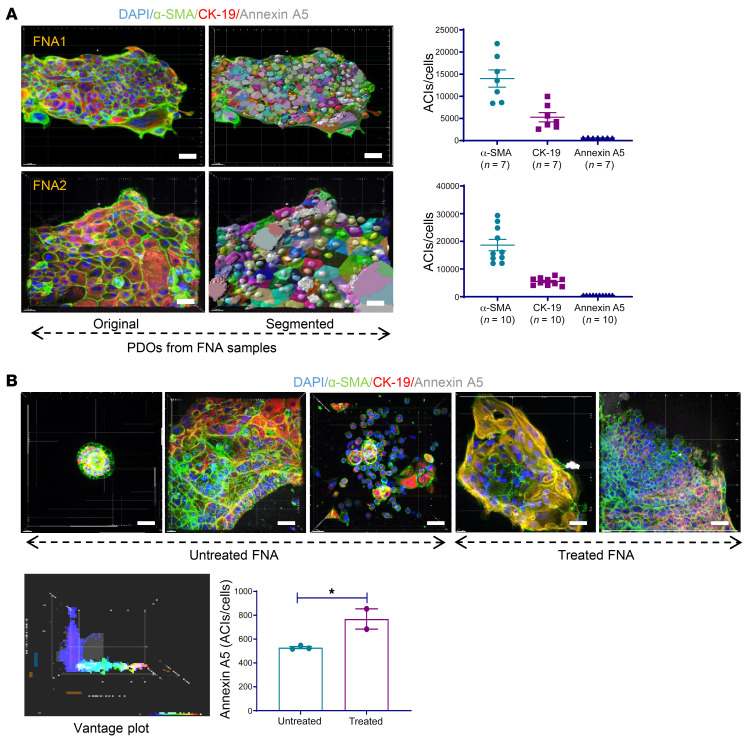
3D imaging analysis in patient-derived organoids generated from endoscopic ultrasound/fine-needle aspiration and surgical pancreatic ductal adenocarcinoma specimens. (**A**) 3D imaging analysis of patient-derived organoids (PDOs) from endoscopic ultrasound/fine-needle aspiration (EUS/FNA) samples. FNA1 original and analysis images and corresponding average ACIs of α-smooth muscle actin (α-SMA), cytokeratin 19 (CK-19), and annexin A5 (top) as well as FNA2 original and analysis images and average ACIs of α-SMA, CK-19, and annexin A5 (bottom). (**B**) Comparison of ACIs for annexin A5 in EUS/FNA PDOs between untreated and treated groups. Five original images from EUS/FNA PDOs are shown, as well as vantage plot of 2 groups of pretreated and untreated PDOs and ACIs for annexin A5. Data are shown as the mean ± SEM by GraphPad Prism 9.0 and statistically analyzed through unpaired *t* test. *P < 0.05. Scale bar: 20 μm.

**Figure 7:**
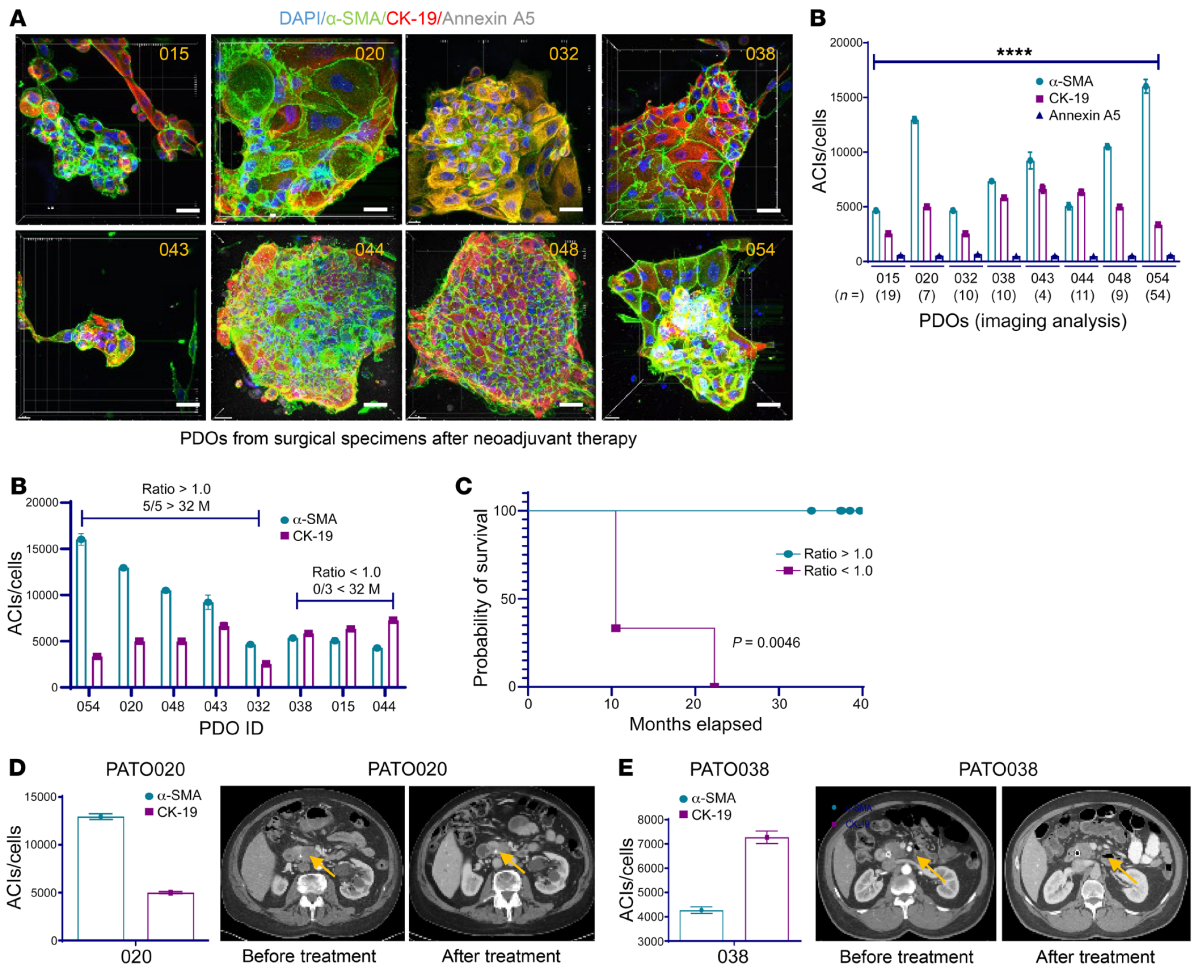
Quantification of imaging analysis in 8 surgical patient-derived organoids from patients who had neoadjuvant therapy. (**A**) Original images of the patient-derived organoids (PDOs) and ACIs for α-SMA, CK-19, and annexin A5 (scale bar: 20 μm). Significant differences were found in each PDO according to 1-way ANOVA. ****P < 0.0001. (**B**) Quantification and ratios of α-SMA and CK-19 in 8 PDOs, ratio > 1.0 versus < 1.0 (*P* = 0.0179, Fisher’s exact test). (**C**) Overall survival curve by Kaplan-Meier (*P* = 0.0046). (**D**) ACI for α-SMA and CK-19 in PATO020 and computed tomography scans before and after treatment. (**E**) ACI for α-SMA and CK-19 in PATO038 and computed tomography scans before and after treatment. Data are shown as the mean ± SEM by GraphPad Prism 9.0.

Establishing PDOs and performing 3D imaging quantifications using ex vivo ODSA with EUS/FNA tumor samples may provide useful information about anticancer drug sensitivity and aid in the selection of upfront chemotherapy. The cytoplasmic algorithm was employed for PDOs from EUS/FNA specimens, and IF staining was used for PDOs derived from surgical specimens. Two representative PDOs from EUS/FNA samples were chosen from 3D imaging analysis. The ACIs for α-SMA, CK-19, and annexin A5 in 7 different PDOs in 1 PDO (FNA1) are shown (Figure 6A, top). The ACIs for α-SMA, CK-19, and annexin A5 in 10 different PDOs in another PDO (FNA2) are shown (Figure 6A, bottom). Annexin A5 levels in treated and untreated EUS/FNA PDOs were compared, demonstrating higher annexin A5 ACIs in treated EUS/FNA PDOs than in untreated PDOs (*P* < 0.05), as indicated in the vantage plot and graph in Figure 6B. No significant ACI differences were observed for α-SMA or CK-19 levels in these groups, indicating a possible correlation between annexin A5 and patient tumor responses. Because differences in α-SMA and CK-19 levels were noted throughout the development of 3D imaging approaches to quantify apoptosis following treatment, we next examined whether the relative cellular composition of PDAC organoids might correlate with outcomes of the patients from which the PDOs were derived. Eight PDOs from treated PDAC surgical samples were subjected to 3D imaging analysis. The ACIs for α-SMA, CK-19, and annexin A5 were significantly different in each PDO (*P* < 0.0001; Figure 7A). Further analysis of the ACIs of α-SMA and CK-19 for these PDOs indicated that patients with α-SMA/CK-19 level ratios of more than 1.0 were associated with improved outcomes and PDOs exhibiting α-SMA/CK-19 ratios of less than 1.0 were associated with worse outcomes. Specifically, patients from whom PDOs PATO054, PATO020, PATO048, PATO043, and PATP032 were derived (α-SMA/CK-19 ratios > 1) were still alive after 32 months, but no patients were living after 32 months in the group with α-SMA/CK-19 ratios of less than 1.0 (Figure 7B, *P* = 0.0179). Accordingly, overall survival for patients with PDO α-SMA/CK-19 ratios of more than 1 was higher than for patients with PDOs α-SMA/CK-19 ratios of less than 1 by Kaplan-Meier analysis (Figure 7C, *P* = 0.0046). These results were supported by clinical objective tumor responses; for example, in PATO020 containing a α-SMA/CK-19 ratio of more than 1.0, radiographic reductions in tumor size on computed tomography imaging during neoadjuvant treatment occurred after 5 cycles of FFX (3.5 × 2.8 cm to 1.4 × 2.3 cm, 74.2% tumor volume reduction; Figure 7D). In comparison, PATO038 exhibited a α-SMA/CK-19 ratio of less than 1.0 and exhibited no substantial radiographic response after 8 cycles of FFX (3.2 × 2.8 cm to 3.1 × 2.7 cm; Figure 7E). Although the examined cohort sample sizes are small, our initial work indicate that treated stromal responses in the ODSA are heterogenous but correlate with overall patient survival (35, 36). We performed a similar analysis using a FFPE tissue microarray (TMA) comprising 99 patients with PDAC and found no correlation between α-SMA/CK-19 ratios and patient survival (Supplemental Figure 7C). Additional analysis of tumor-stroma modulation in treated PDAC tumors derived from larger patient cohorts will be necessary to validate our findings and to confirm potentially prognostic roles of α-SMA/CK-19 ratios in treated PDOs.

## Discussion

A standard-of-care-therapy-fits-all approach is unlikely to significantly improve PDAC outcomes. Rather, a personalized and patient-specific approach, complete with independent platforms capable of assessing antitumor and tumor-stroma cell responses in individual patients, is likely to be required (30, 37). We therefore developed an OBP that combines 3D organoid imaging quantification with 3D 96-well plate drug screening through colorimetric readouts of cell viability (ex vivo ODSA) to evaluate drug efficacy responses for individual patients with PDAC. The OBP required substantially less time and fewer resources than other assays (Figure 2 and Supplemental Figure 4) on the 3D platform, and we were able to assess in individual patients the total number of cells and nuclei as well as quantify multiple antigens for segmentation of tumor-cell targeting or specific stromal proteins on a 3D diagram. In our system, quantification of Ki-67 intensity in PDOs showed no alterations of ANI for Ki-67 from day 7 to day 14, which allowed for objective use of the OBP to assess 3D IF imaging analysis and antitumor efficacy over 4–14 days, which is more efficient than an in vitro model (Figure 2). The OBP results in 5–7 days, and ex vivo ODSA can be applied for *personalized* medicine when 3D confocal microscope is not required (Figure 3E). During these days of tumor tissue acquisition, the OBP could generate results in a timeline compatible with standard patient management while potentially informing the selection of chemotherapy. Overall CK-19 levels decreased over time, with signals localizing to the periphery of visualized organoids (Figure 1 and Figure 2E), which indicated reductions of CK-19 within deeper regions of PDAC organoids; more studies are needed to verify this phenomenon and determine whether it affects potential drug responses. Tumor-stroma modulation showed critical influence on antitumor efficacy in individual patients (Figure 7, B, C, and E), indicating distinct clinical outcomes among patients. Thus, our model system favorably reproduces the heterogeneity of patient tumors and corresponding responses to chemotherapy.

The OBP analysis showed variations in antitumor and tumor-stroma modulation both in vivo and ex vivo that influenced the efficacy of drugs in individual PDOs after neoadjuvant regimens. The ex vivo ODSA results when compared with CA19-9 status demonstrated predictive ODSA screening for clinical regimens that could be used to direct treatment guidelines and strategies for PDAC. The current study therefore demonstrates the feasibility of using an OBP to select chemotherapeutic regimens for individual patients and the predictive capability of an OBP for personalized clinical decision-making and/or personalized medicine as well as for screening novel drugs or regimens for PDAC in prospective clinical trials. Our OBP can be applied to both surgical and EUS/FNA tumor specimens, which extends its application to patients with all stages of PDAC (Figure 6A). Patients with high α-SMA or α-SMA/CK-19 ratios of more than 1.0 appeared to have better treatment responses and outcomes, suggesting that the α-SMA/CK-19 ratio could be used as a novel predictive factor for PDAC. To the best of our knowledge this has not been previously reported in the literature, and additional analysis involving with more patients and data sets is needed to validate our findings. Although correlations between α-SMA/CK-19 ratios and patient survival in PDOs were not supported by the PDAC FFPE TMA tested in our study, we believe that the disaggregation of tumors into single-cell suspensions, followed by their reaggregation in varying proportions of tumor cells and fibroblasts as PDOs in 3D culture, is vastly different from FFPE and represents a functional assay reflective of underlying cancer biology. As such, we do not believe that null findings with respect to α-SMA/CK-19 ratios observed in FFPE PDAC tumors detract from the potential prognostic ability of α-SMA/CK-19 ratios in disaggregation assays/PDO formation.

Cells that comprise PDOs may exhibit varying levels of CK-19 and/or α-SMA during ex vivo culturing or passaging (Supplemental Figure 3). So far no reports have explained this phenomenon, which could explain why stromal subtype analysis is needed to determine antitumor efficacy in an in vitro model (35). This phenomenon suggests that optimization of PDO culture is crucial, especially for PDOs from patients who have received neoadjuvant therapy, to prevent PDOs from losing cells or stopping growth during passages. Our findings suggest that pairing known genomic alterations with specific anticancer therapy could allow molecular profiling for individual patients with PDAC, and additional studies exploring molecular profiling in patients with PDAC are needed.

Taken together, our findings demonstrated that the OBP can be used concurrently for IF imaging quantification and an antitumor drug sensitivity assay in 3D format. Our system expands the use of a PDAC organoid model in translational cancer research for antitumor efficacy and tumor-stroma modulation; quantifications of α-SMA and CK-19 could be used to predict patient clinical outcomes and develop preclinical novel drug trials. In addition, the OBP can directly investigate resected and EUS/FNA human tumor samples to assess the activity of multiple therapeutic regimens, allowing clinicians to get information about the regimens and tumor-stroma modulation in a timely manner. We expect this platform, which we believe to be novel, to eventually be applied to personalized cancer treatment as an independent approach or in combination with molecular biomarker-guided strategies. In addition, 3D image analysis and the ex vivo ODSA are applicable to large-scale drug screening with the same algorithms in group analysis of clinical trials in future studies.

## Methods

### Patient specimens.

This retrospective analysis was performed at The University of Texas MD Anderson Cancer Center. Patient tumors were collected only after planned surgical resection and pathologic examination. A total of 321 patient samples were obtained from pancreatectomies performed from 2009 to 2019, and FNA tumor samples were obtained from 39 patients beginning in 2018. FFPE tumors from 99 patients with PDAC who underwent surgical resection were organized into a TMA (2 cores per patient). All tumor specimens obtained at MD Anderson were selected after reviewing patient medical records and tissue samples.

### Establishment of organoids from PDXs and surgical and EUS/FNA samples, ex vivo ODSA, and reagents.

PDAC PDXOs were initially established in at least triplicate following the establishment of optimized conditions of 3D culture and IF staining. All organoid growth media were the same for PDXOs and human samples and have been described previously (19, 20). Briefly, tumors were digested in Dispase II and collagenase type II (surgical or EUS/FNA samples) or IV (PDX samples), washed, and seeded in a 24-well plate with Matrigel. The ODSA was established using a similar procedure: organoids were immediately generated upon acquisition of tumor tissue (day 0), allowed to enter a growth phase (days 0–2), and treated with standard-of-care drug regimens on days 3–5 with organoid grown media (OGM) to ultimately evaluate drug efficacy and IF imaging analysis on days 7–14 (Supplemental Figure 4). Cells were digested, counted, and seeded in 96-well plates (3,000–5,000 cells per well) in triplicate. At defined intervals, after exposure to antitumor drugs, cell viability was read using commercial reagents (20, 30) with the FLUOstar Omega microplate reader (BMG LABTECH). The CellTiter-Glo 3D Cell Viability Assay was purchased from Promega (catalog G9681). Reagents for organoid establishment and culture were Dispase II, collagenase type IV, and collagenase type II (Thermo Fisher Scientific; catalog 17105-041, 17101-015, and 17104-019, respectively); growth factor–reduced Matrigel (Corning; catalog 356231); and DNase I (Sigma-Aldrich; catalog D5025-150KU). Organoid growth medium components included advanced Dulbecco’s modified Eagle medium/F-12 medium, GlutaMAX supplement, and HEPES (1 M), all obtained from Thermo Fisher Scientific (catalog 12634010, 35050061, and 15630080, respectively). Recombinant human epidermal growth factor protein, recombinant human fibroblast growth factor-10 protein, recombinant human Noggin protein, recombinant human R-spondin 1 protein, gastrin I (human), Y-27632 dihydrochloride, A83-01, and Nicotinamide were purchased from R&D Systems (catalog 236-EG-200, 345-FG-025, 6057-NG-025, 4645-RS-025, 3006/1, 1254/1, 2939/10, and 4106/50, respectively). Nicotinamide was purchased from Sigma-Aldrich (catalog A9165-5G). Primocin was purchased from InvivoGen (catalog ant-pm-1). L Wnt-3A cells were obtained from the ATCC (catalog CRL-2647). Cultrex HA-R-spondin 1-Fc 293T cells were purchased from R&D Systems (catalog 3710-001-01). Wnt-3A–conditioned media was collected following the vendors’ instructions and was frozen in preparation for OGM (19). AUR (Sigma-Aldrich; catalog A6733), a gold complex that induces significant apoptosis by increasing annexin A5 expression (38, 39), was used as a positive toxicity control for the ex vivo ODSA (15). Antibodies for IF and Western blot are listed in Supplemental Table 6. All organoid culturing was maintained in OGM at 37°C in a 5% CO_2_ environment unless stated otherwise, as instructed by the vendors. All imaging analysis and ODSA on PDOs in this study was completed in passage 2 unless specified otherwise.

### Protocols for 3D confocal microscopy and Imaris software analysis.

Organoids grew directly in 8-well chamber slides (Ibidi; catalog 80826) for 3 days and were exposed to GEM at different doses, as shown in the Supplemental Table 3. Organoid IF staining in the chambers was performed after 4 days of treatment. Organoid 3D serial images were captured using an Andor Revolution XDi WD Spinning Disk Confocal microscope (Olympus America) in the MD Anderson Flow Cytometry and Cellular Imaging facility. This microscope supports simultaneous multichannel imaging and a wide information bandwidth. Available excitation wavelengths used in the study included 405, 488, 594, and 647 nm. Acquired serial confocal *Z*-stacks of 5 μm were captured and reconstructed into 3D in multiple organoids each for control and treatment conditions and analyzed using the Imaris Bitplane 9.2 software program (Oxford Instruments). Average nuclei or cytoplasmic intensities were calculated though all organoid nuclei or cell numbers. 3D organoids were chosen at depth ranges between 40 and 120 μm.

### 3D and IF image quantification algorithms.

Imaris Arena provided sets of features for visualization and analysis of multiple channels of IF data sets for static 3D organoid images. Cytoplasmic and nuclear algorithms for image processing, segmentation, classification, and reporting were developed following the manufacturer’s instructions. TMAs were scanned and quantified using Visiopharm software.

### IF staining of PDXOs, PDOs, and FFPE TMAs.

PDAC organoids were seeded in 8-well chambers, serially diluted doses of GEM were added to each well (Supplemental Table 3), and IF staining was initiated after 4 days of treatment with GEM. Multiple channels of IF staining were used as described previously (40). IF staining for annexin A5 was performed using a 2-step protocol, because fixation can be disrupted on the cell membrane and expose phosphatidylserine to the antibody, which can cause nonspecific binding of annexin A5 to phosphatidylserine on the inner surface of the cell membrane (41). Briefly, organoids in the chamber were gently washed with PBS and then blocked with 0.1% bovine serum albumin for 20 minutes. Annexin A5 (1:75) was added to each well, and the chamber was incubated overnight at 4°C. The next day, each chamber was washed carefully with PBS, each well was fixed with formalin for 10 minutes and 1% Triton X-for 10 minutes, and then primary antibodies against α-SMA and CK-19 (1:100) were added to the chambers at specific concentrations overnight at 4°C. On the third day, the chambers were washed once with PBS, DAPI (D8417; Sigma-Aldrich) was added for count staining to each well for 10 minutes, and the chambers were filled with PBS. These organoids were scheduled for capture of images. Other primary antibodies except anti–annexin A5 were processed using a 1-step staining protocol. As a negative control, chambers were incubated with IgG instead of primary antibodies (Supplemental Figure 2B). For TMAs, slides were subjected to antigen retrieval and stained with α-SMA (1:100) and CK-19 (1:100) using a 1-step protocol. All images were obtained at the same exposure time. These profiles of images were then used to discriminate the individual colors on multistained channels. Multiple random images were chosen to estimate ANI and ACI values (×40 magnification).

### Western blot analysis of organoids.

Organoid lysates were prepared for Western blot analysis by collecting growing organoids in 24-well plates after exposing them to 3 doses of GEM. Matrigel domes in each well were broken down and collected with cold PBS with proteinase inhibitor; these samples were spun down, and 50 μL RIPA buffer was mixed with the organoid pellet. Organoid lysates (20 μg) were separated via electrophoresis on 8%–12% SDS polyacrylamide gels, transferred to polyvinylidene fluoride membranes (GE Healthcare; 10600023), and probed with different dilutions of antibodies of interest. The antibodies used for Western blot analysis included anti–BCL-XL and anti–cleaved caspase-3 (Supplemental Table 6). Anti-vinculin was used as a protein loading control for Western blot analysis. Reactive bands were visualized using enhanced chemiluminescent reagents (GE Healthcare).

### Organoid growth curve and the ex vivo ODSA.

The PDXO growth curve was set up using 5,000 cells seeded with Matrigel in a 96-well assay plate (Corning; catalog 3340) in triplicate, and 200 mL of organoid growth medium was added to each well. The plate was read using CellTiter-Glo 3D cell viability reagent (Promega; catalog G9683) every other day following the vendor’s instructions. The ex vivo ODSA treatment time was based on results of PDXO growth curves. Organoids were cultured in 96-well plates in triplicate for 3 to 4 days and exposed to planned doses of chemotherapeutic drugs for 4 more days, and their viability was read using the reagents by the FLUOstar Omega reader (BMG Labtech). The antitumor agents used in the study were purchased from Selleckchem.com: GEM-based regimen GEM HCL (catalog S1149), PAC (catalog S1150), and cisplatin (catalog S1166) and the FFX regimen of 5-fluorouracil (catalog S1209), leucovorin (catalog S1236), irinotecan (catalog S2217), and oxaliplatin (catalog S4128). All of these agents’ stock solutions were prepared following the vendor’s instructions. The values of each well readout were plotted using the Prism 9.0 software program (GraphPad Software).

### Statistics.

All quantified data were plotted and analyzed using Prism 9.0. Significance was determined using the Student’s unpaired 2 tailed *t* test, 1-way ANOVA, and exact McNemar’s test. Data are representative of at least 3 independent experiments and reported as mean ± SEM, unless otherwise indicated. *P* values of less than 0.05 were considered significant. Differences in patient survival based on α-SMA/CK-19 ratios were tested using the Kaplan-Meier log-rank test.

### Study approval.

The PDAC PDX protocol was approved by the MD Anderson Institutional Review Board (no. LAB07-0854). Acquisition of PDAC specimens from FNA biopsies was approved by the MD Anderson Institutional Review Board (no. LAB00-0396). Informed consent was obtained from each patient.

## Author contributions

YK and MPK designed research studies. JD, JL, XL, HW, and JKB conducted experiments. MSB, BRW, RAW, NI, CWDT, JEL, MHGK, JHL, and MPK provided reagents. YJC provided statistics analysis. EJK and MWH acquired data. YK and MPK wrote the manuscript. YK, JBF, and MPK analyzed data. PJC, AM, JEL, RAD, SP, FM, JBF, and MPK revised the manuscript.

## Supplementary Material

Supplemental data

ICMJE disclosure forms

Supplemental video 1

Supplemental video 2

Supplemental video 3

## Figures and Tables

**Table 1:**
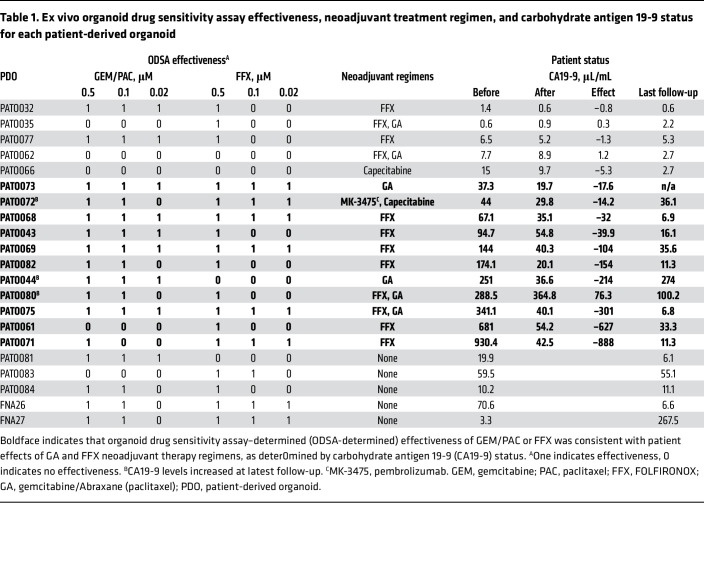
Ex vivo organoid drug sensitivity assay effectiveness, neoadjuvant treatment regimen, and carbohydrate antigen 19-9 status for each patient-derived organoid

## Version 1 10/25/2022

In-Press Preview

## Version 2 12/15/2022

Electronic publication
