# Supplementary material for: 3D imaging analysis on an organoid-based platform guides personalized treatment in pancreatic ductal adenocarcinoma
Source: J Clin Invest. 2022 Dec 15;132(24):e151604. doi: 10.1172/JCI151604 (PMC9753992; doi:10.1172/JCI151604)
Supplement: Supplemental data [file jci-132-151604-s094.pdf]

Supplementary Data

Supplementary tables

Supplementary Table S1. Patient clinicopathologic features corresponding to the patient-derived xenograft organoids (PDXOs) used in the study [A]

PDXO	Sex [B]	Age /Year	Race /ethnicity	Neoadjuvant /regimens [C]	Tumor /site	AJCC [D]	Differentiation
PATXO 032	M	63	White	Gem + erlotinib	Head	IIA	Moderate
PATXO 034	M	61	White	GA	Head	IIA	Moderate
PATXO 039	M	62	White	None	Head	IIA	Poor
PATXO 043	M	71	White	GEM + XRT [E]	Tail	IB	Poor
PATXO 045	M	50	White	None	Body	IIB	Moderate
PATXO 046	F	82	White	None	Body	IIA	Moderate
PATXO 050	F	60	White	None	Head	IIB	Moderate
PATXO 053	M	66	White	Xeloda [F] + XRT	Liver	IV	n/a
PATXO 055	F	64	Black	None	Head	IIB	Poor
PATXO 056	M	61	White	Gem + Cis [G]	Head	IIB	Poor
PATXO 060	M	81	White	None	Head	IIB	Moderate

PATXO 066	M	63	White	None	Head	IIB	Moderate
PATXO 069	M	67	White	None	Tail	IIA	Poor
PATXO 070	F	55	White	Gem + Cis	Body	IIB	Poor
PATXO 076	M	57	White	Gem + XRT	Lung	IV	Moderate
PATXO 079	M	60	White	Gem + XRT	Head	IIB	Moderate
PATXO 087	M	75	White	Xeloda + XRT	Head	IIA	Moderate
PATXO 118	F	71	White	None	Tail	IIB	Poor
PATXO 124	F	63	White	FFX	Head	IIB	Moderate
PATXO 148	M	74	White	None	Liver	IV	Poor
PATXO 153	F	57	White	FFX, Gem + XRT	Liver	IV	Poor
PATXO 179	M	52	Hispanic	None	Liver	IV	Poor
PATXO 216	M	85	White	n/a	Head	IIB	Moderate
PATXO 245	M	49	White	None	Liver	IV	Poor
PATXO 248	M	46	White	None	Liver	IV	Poor

PATXO 254	M	71	White	FFX	Head	IIB	Moderate
PATXO 262	F	62	White	None	Neck	IIB	Moderate
PATXO 274	F	74	White	GA	Head	IIB	Poor
PATXO 290	M	66	White	FFX, GA	Neck	IIB	Poor
PATXO 296	F	71	White	FFX	Tail	IIB	Moderate
PATXO 301	F	65	White	FFX, GA	Head	IIB	Moderate
PATXO 304	M	59	White	GA	Head	IIA	Moderate
PATXO 308	F	72	Black	None	Head	IB	Moderate

[A] All of these patients had a confirmed diagnosis of pancreatic ductal adenocarcinoma, and PDOs were from surgical specimens or endoscopic ultrasound/fine-needle aspiration specimens. [B] M, male; F, female. [C] FFX, FORFIRONOX; GA, gemcitabine/Abraxane (paclitaxel). [D] American Joint Committee on Cancer stage, version 7. [E] Radiotherapy. [F] capecitabine. [G] Cisplatin.

Supplementary Table S2. Details of each organoid for figures

Figure		PDXO/PDO (passage 2)	# unique examined organoids	Organoid thickness (µm)	Cell numbers per organoid	Average cells per organoid
Fig. 1	1C, D	PATXO118	8	92	338;235;319;250;391;534;93;178	292.2 ± 135.6
	1F, G	PATO044 (P2)	11	143	104;93;112;382;129;60;49;419;146;17;199	155.4 ± 30.9
Fig. 2	2C Day 7	PATXO296	17	143	164;111;130;127;138;125;129;143;87;97;87;123;124;95;131;96;123	119.4 ± 21.1
	2D Day 14	PATXO296	19	153	295;270;94;345;329;448;262;264;129;233;218;289;190;189;218;266;99;320;345	252.7 ± 89.5
Fig. 3	3C Vehicle	PATXO066	12	77	135;197;169;70;126;102;120;268;196;202;177;273	169.5 ± 62.5
	3C AUR 0.76	PATXO066	12		137;86;32;74;110;215;135;119;163;70;62;125;	110.5 ± 50.1
	3C AUR 7.6	PATXO066	12		124;137;171;56;130;31;111;64;65;69;73;77	92.3 ± 41.2
	3C AUR 10.0	PATXO066	10		59;39;27;29;10;60;26;8;13;52	32.3 ± 19.5
Fig. 4	4C vehicle	PATO020	7	92	11;12;25;6;38;3;3	14.0 ± 13.0
	4C GEM 0.16	PATO020	7		10;14;9;3;3;7;28	10.5 ± 8.6
	4C GEM 0.8	PATO020	8		10;11;14;9;7;17;8;9	10.6 ± 3.3
	4C GEM 4.0	PATO020	8		13;7;9;37;25;18;20;6	16.8 ± 10.5
	4H vehicle	PATO015	19	104	76;62;59;73;102;68;43;57;36;52;69;46;65;79;70;106;55;61;43	64.3 ± 18.3
	4H GEM 0.16	PATO015	10		32;102;70;18;41;67;155;52;135;14	68.6 ± 48.2
	4H GEM 0.8	PATO015	23		34;30;17;11;13;14;27;18;49;12;36;17;20;34;27;20;24;25;18;14;21;26;49	24.1 ± 10.6
	4H GEM 4.0	PATO015	12		17;21;22;19;11;20;20;15;23;31;15;13	18.9 ± 5.3

Fig. 5	5A-FNA1	FNA16	7	57	442;401;650;327;528;190;230	395.4 ± 162.7
	5A-FNA2	FNA27	10	104	29;26;211;11;336;158;256;181;95;29	132.9 ± 113.2
	5B	FNA26	9	44	6;7;32;22;7;7;7;5;19	12.4 ± 9.5
	5B	FNA27	10	104	29;26;211;11;336;158;256;181;95;29	132.9 ± 113.2
	5B	FNA31	1	86	216	216
	5B	FNA24	5	40	10;8;41;83;106	49.6 ± 43.7
	5B	FNA16	7	57	442;401;650;327;528;190;230	395.4 ± 162.7
	5C-015	PATO015	19	104	59;47;42;42;72;53;37;39;41;45;48;35;50;50;47;72;39;37;38	47.0 ± 10.7
	5C-020	PATO020	7	92	6;3;12;25;38;4;11	14.1 ± 12.8
	5C-032	PATO032	10	64	26;22;18;39;20;25;18;38;41;33	28.0 ± 8.9
	5C-038	PATO038	10	182	92;52;248;50;63;102;236;128;94;373	143.8 ± 106.8
	5c-043	PATO043	4	58	20;23;23;39	26.2 ± 8.6
	5C-044 (P2)	PATO044	11	143	129;93;104;60;112;382;49;199;17;146;419	155.4 ± 30.9
	5C-048	PATO048	9	113	52;44;44;51;42;56;50;36;55	47.7 ± 6.6
	5C-054	PATO054	12	84	15;26;8;12;26;30;17;15;10;28;31;11	19.0 ± 8.5
Fig. S1	S1A	PATO044 (P2)	11	143	104;93;112;382;129;60;49;419; 146;17;199	155.4 ± 30.9
Fig. S2	S2A	PATXO296	17	143	164;111;130;127;138;125;129;143;87;97;87;123;124;95;131;96;123	119.4 ± 21.1
Fig. S3	S3A	PATO044 (P2)	11	143	104;93;112;382;129;60;49;419; 146;17;199	155.4 ± 30.9
		PATO044 (P4)	11	181	93;100;342;349;164;166;133;59;136;132;106	161.8 ± 95.9
		PATO044 (P7)	9	78	88;76;30;83;26;55;51;57;63	58.7 ± 21.6
Fig. S4	S4A	PATXO066 V	6	155	15;6;6;8;5;9	8.1 ± 3.6
		PATXO066 G0.16	7		16;12;22;37;38;16;16	22.4 ± 10.7
		PATXO066 G0.8	10		38;5;5;12;22;12;22; 13;13;10	15.2 ± 9.8
		PATXO066 G4.0	8		8;3;5;8;8;6;10;21	8.3 ± 5.6
	S4E	PATXO118 V	8	91	14;70;15;11;14;15;8;16	20.3 ± 20.2
		PATXO118 G0.16	10		18;20;19;20;16;19;18;16;16;18	18.0 ± 1.5

		PATXO118 G0.8	9		8;29;14;21;14;18;21;29;6	17.7 ± 8.1
		PATXO118 G4.0	9		17;9;15;13;15;6;10;20;6	12.3 ± 4.8

Supplementary Table S3. Gemcitabine (GEM)-based doses used in the *ex vivo* organoid drug sensitivity assay [A]

Dose	GEM (µM)	PAC (µM)	CIS (µM)
High	4.0–0.5	1.0–0.5	4.0
Medium	0.8–0.1	0.2–0.1	0.8
Low	0.16–0.02	0.04–0.02	0.16

[A] High dose was adjusted to 0.5 µM in patient-derived organoids for the GEM/PAC combinations. PAC, paclitaxel; CIS, cisplatin.

[B] GEM was combined with each drug in three serial diluted doses.

Supplementary Table S4. FOLFIRONOX (FFX) doses used in the *ex vivo* organoid drug sensitivity assay [A]

	FFX (µM) [B]			
**Dose**	**5-FU[C]**	**Leucovorin**	**Irinotecan**	**Oxaliplatin**
High	4.0–0.5	4.0–0.5	2.0–0.5	1.0–0.5
Medium	0.8–0.1	0.8–0.1	0.4–0.1	0.2–0.1
Low	0.16–0.02	0.16–0.02	0.08–0.02	0.04–0.02

[A] High dose was adjusted to 0.5µM in patient-derived organoids for the FFX regimens.

[B] FFX was combined with each drug in three serial diluted doses.

[C] 5-FU, fluorouracil.

Supplementary Table S5. Patient clinicopathologic features corresponding to the patient-derived organoids (PDOs) used in the study [A]

PDO	Sex [B]	Age, years	Race/ethnicity	Neoadjuvant regimens [C]	Tumor site	AJCC [D]	Differentiation
PATO015	M	72	White	FFX	Head	IIA	Poor
PATO020	M	66	White	FFX	Body	IB	Moderate
PATO032	M	61	Black	FFX	Head	n/a	Moderate
PATO038	M	56	White	FFX	Head	IIB	Poor
PATO043	M	72	Asian	FFX	Head	IIA	Moderate
PATO044	F	79	White	GA	Head	IIB	Poor
PATO048	F	71	White	FFX	Tail	IIB	Moderate
PATO054	M	73	White	FFX	Head	IIB	Moderate
PATO061	M	48	White	FFX	Head	IIB	Poor
PATO062	F	61	White	FFX, GA	Head	IIB	Moderate
PATO066	F	61	White	Capecitabine	Head	IIB	Moderate
PATO068	F	58	White	FFX	Head	IIB	Moderate
PATO069	M	71	Asian	FFX	Head	IIB	Moderate
PATO071	M	71	White	FFX	Head	IIB	Moderate
PATO072	M	62	White	Capecitabine, MK-3475[E]	Neck	IIB	Poor
PATO073	F	65	White	GA	Head	IIB	Poor
PATO075	M	66	White	FFX, GA	Neck	IIB	Poor
PATO077	M	51	White	FFX	Head	IIB	Moderate
PATO080	M	68	White	FFX, GA	Body	IIB	Moderate
PATO081	F	66	White	None	Head	IIA	Moderate
PATO082	F	67	White	FFX	Head	IB	Moderate
PATO083	F	67	White	None	Neck	IIB	Moderate
PATO084	M	81	White	None	Tail	IB	Moderate
FNA016	M	75	White	FFX	Head	n/a	n/a
FNA024	M	65	Black	GA	Body	III	n/a
FNA026	F	56	White	None	Tail	n/a	n/a
FNA027	F	77	White	None	Head	n/a	n/a
FNA031	M	66	White	None	Head	IIB	Poor

[A] All of these patients had a confirmed diagnosis of pancreatic ductal adenocarcinoma, and PDOs were from surgical specimens or endoscopic ultrasound/fine-needle aspiration specimens. [B] M, male; F, female. [C] FFX, FORFIRONOX; GA, gemcitabine/Abraxane (paclitaxel). [D] American Joint Committee on Cancer stage. [E] Programmed cell death protein 1 inhibitor.

Supplementary Table S6. List of antibodies used in this study

Name	Vendor	Category #
anti-annexin A5 Alexa Fluor 647	Santa Cruz Biotechnology	sc-74438AF647
anti-α-SMA Alexa Fluor 488	Abcam	ab202295
anti-CK-19 Alexa Fluor 594	Abcam	Ab203443
anti-Ki-67 Alexa Fluor 594	Cell Signaling Technology	12075
anti-BCL-XL	Cell Signaling Technology	2764
anti-cleaved caspase 3	Cell Signaling Technology	9664
Anti-vinculin	Abcam	ab18058

Supplementary Figure Legends

Supplementary Figure 1. Quantification of 3D imaging analysis in PDXO using nuclear algorithms and Imaris software. (A) Nuclear algorithms were created for 3D imaging; each processing and segmentation step followed the Imaris instructions. All immunofluorescence images shown are 3D view renderings (40 × magnification). (B) Nuclear imaging analysis results showing the total number of cells and nuclei in 17 fields. (C) Ki-67 index (left) and average nuclear intensity for Ki-67 (right). (D) Average cytoplasmic intensities for alpha-smooth muscle actin (α-SMA) and cytokeratin 19 (CK-19). Results are presented as means ± standard error of the mean by GraphPad Prism 9.0.

Supplementary Figure 2. Serial 2D slices images from PATO044 in Figure 2E. (A) Slices from 1 to 115 µm original images; 100% cut pixel (200 µm X 200 µm) at x:y (808 x 274) serial images (Insert). (B) Original images for IgG control in PDXOs of PATXO060 and PATXO069.

Supplementary Figure 3. 3D imaging analysis of a PDO (PDO044) during passages (P) 2, 4, and 7. (A) Original 3D images. (B) Vantage plot of images for three passages. (C) ACIs for α-SMA. (D) ACIs for CK-19. Results are presented as means ± standard error of the mean by GraphPad Prism 9.0. and statistically analyzed through unpaired t test. **P < 0.01. (E) ACIs for Annexin A5.

Supplementary Figure 4. The *ex vivo* ODSA workflow

Supplementary Figure 5. Quantification of GEM-induced apoptosis in patient-derived xenograft organoids after exposure to GEM at different doses. 3D images show the organoids (A) PATXO066 and (E) PATXO118 after treatment with GEM (G) at 0.16 µM, 0.80 µM, and 4.00 µM compared with vehicle control (V). Nuclei were counterstained with DAPI (blue). α-SMA, alpha-smooth muscle actin; CK-19, cytokeratin 19. (B) *Ex vivo* organoid drug sensitivity assay (ODSA) results for PATXO066 read using cell viability for treatment with vehicle control or GEM at 0.16 µM, 0.80 µM, or 4.00 µM for 4 days. **P < 0.01 for 0.16 µM GEM; ****P < 0.0001 for 0.8 µM and 4.0 µM GEM. (C) Average cytoplasmic intensities (ACIs) for annexin A5 in PATXO066, which increased with treatment with 0.8 µM GEM (****P < 0.001) and 4 µM GEM (***P < 0.001). (D) Western blot analysis showing expression of cleaved caspase 3 and B-cell lymphoma-extra large (BCL-XL) in PATXO066 after treatment with GEM at different doses for 4 days. (F) *Ex vivo* ODSA results for PATXO118 read using cell viability for treatment with vehicle control or GEM at 0.16 µM, 0.80 µM, or 4.00 µM for 4 days. ***P < 0.001 for the three doses of GEM. (G) ACIs for annexin A5 in PATXO118. ***P < 0.001 for 4.00 µM GEM. (H) Western blot analysis of PATXO118 showing expression of cleaved caspase 3 and BCL-XL after treatment with GEM at different doses for 4 days. Results are presented as means ± standard error of the mean by GraphPad Prism 9.0 and statistically analyzed through ordinary one-way ANOVA.

Supplementary Figure 6. *Ex vivo* organoid drug sensitivity assay (ODSA) results in PDXOs and PDOs through cell viability readouts. PATXO066 (A) and PATXO118 (B) were exposed to three

doses of GEM)/paclitaxel (PAC) or GEM/cisplatin (CIS) for 4 days. ****P < 0.0001 for the three doses compared with control. (C) PATXO060 and PATXO053 exposed to three doses of FOLFIRONOX (FFX) for 4 days. **P < 0.01 for the low dose in PATXO053 (left); ****P < 0.0001 for the medium and high doses in PATXO053; **P < 0.01 for the three doses in PATXO060 (right). (D) The endoscopic ultrasound/fine-needle aspiration PDO FNA34 was exposed to three doses of GEM/PAC or FFX for 4 days. No significant differences were observed at these doses. (E) Embedded binary PDO responses (1=responder, 0=non-responder) as a function of drug treatment dose (high, medium low) and organoid viability thresholds (10%, 20%, 30%, 40%) in 21 different patient organoid models (PATO or FNA). A red cell denotes a PDO response (responder) after treatment with high, medium, and low drug treatment doses. A yellow cell denotes an intermediate response as qualified by a PDO response to 1-2 drug treatment doses. A green cell denotes no PDO response to all drug treatment doses (non-responder). PDO models in **BOLD** are derived from patients who responded to chemotherapy treatments as measured by CA19-9 reductions. (F) PDO responses distilled into a binary response grid. A red cell denotes a PDO response (responder) as qualified by a PDO response to 1-2 drug treatment doses. A green cell denotes no PDO response to all drug treatment doses (non-responder). PDO models in **BOLD** are derived from patients who responded to chemotherapy treatments as measured by CA19-9 reductions. The blue cell denotes a responder model, consistent with patient tumor response, at ≤30% threshold but not at the 40% threshold. *PATO0080 (asterisk) responded to drug treatments in the ODSA but did not match with patient tumor response.

Supplementary Figure 7. Global PDO responses to drug treatment at a 30% treatment response threshold. (A) Twenty-one PDOs from unique patients were treated with increasing doses of GEM/PAC or (B) FFX. Solid horizontal lines represent 100% control and dashed horizontal lines represent 30% viability reduction thresholds. Results are presented as means ± standard error of the mean in triplicate by GraphPad Prism 9.0 and statistically analyzed through ordinary one-way ANOVA. (C) Multi-color immunofluorescent quantitation and analysis of α-SMA/CK-19 ratios were performed in 99 patient PDAC tumors arranged in a FFPE TMA. No significant survival differences were observed between two patient groups with α-SMA/CK-19 ratios greater or less than 1 (Kaplan-Meier log-rank test, $p=0.249$).

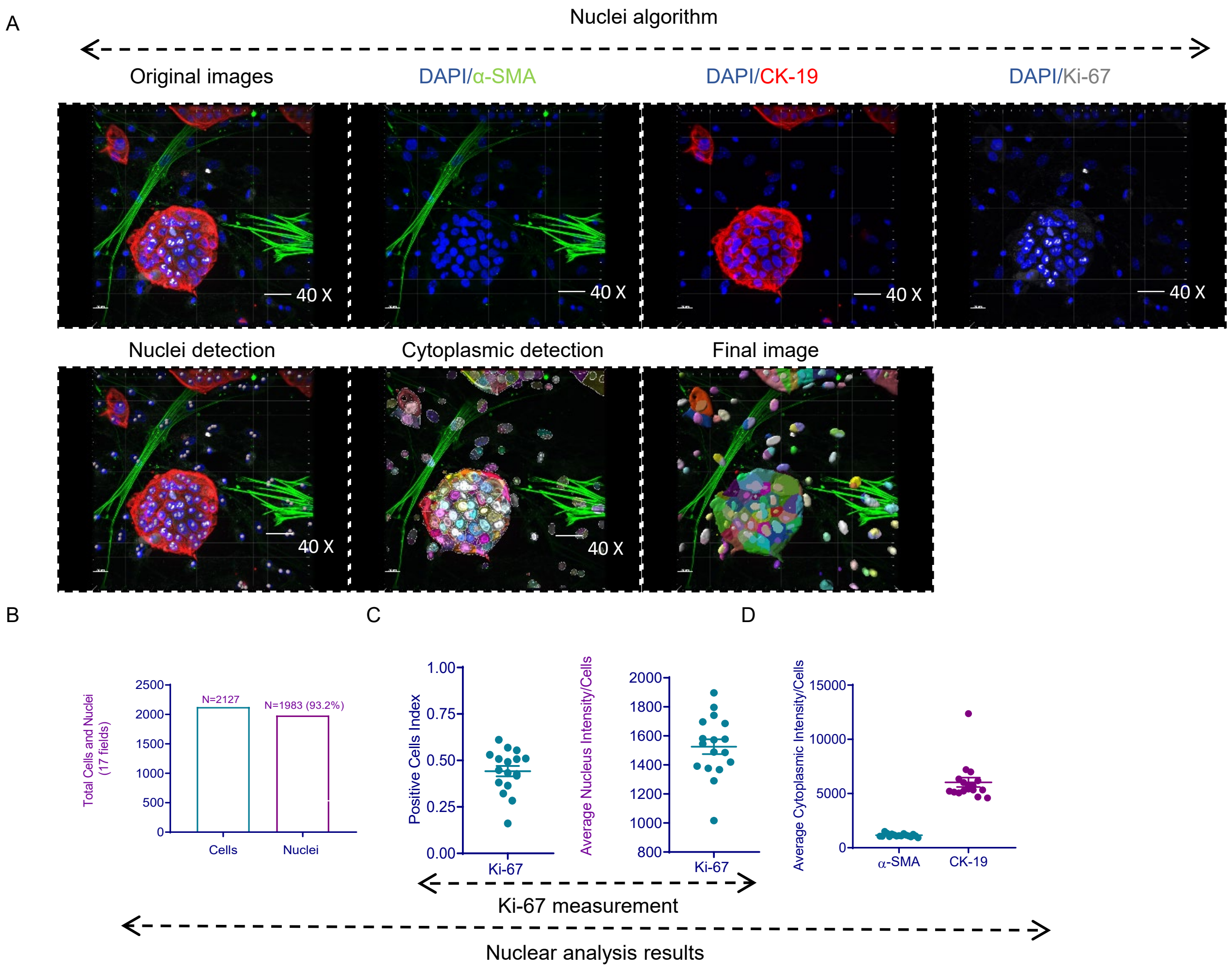

Supplementary Figure 1

A

PATO044 slices from 1 to 60 µm

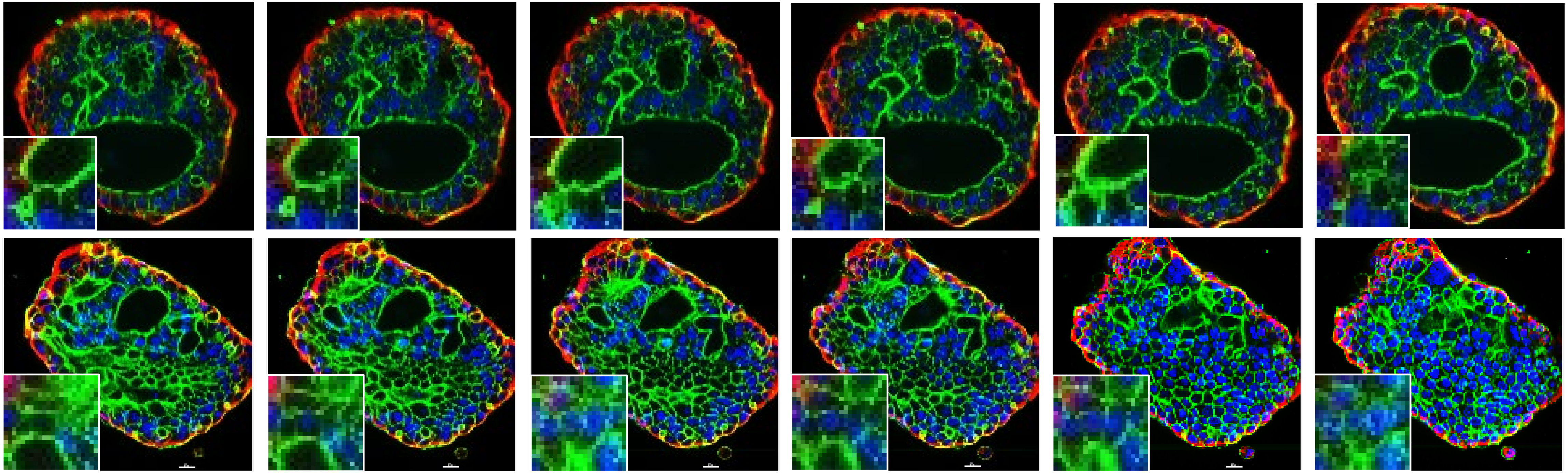

PATO044 slices from 65 to 115 µm

B

IgG control

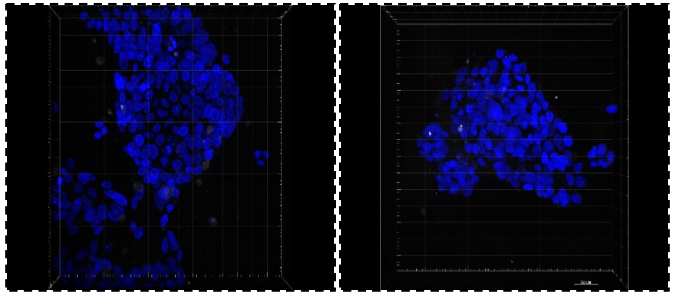

PATXO060 PATXO069

Supplementary Figure 2

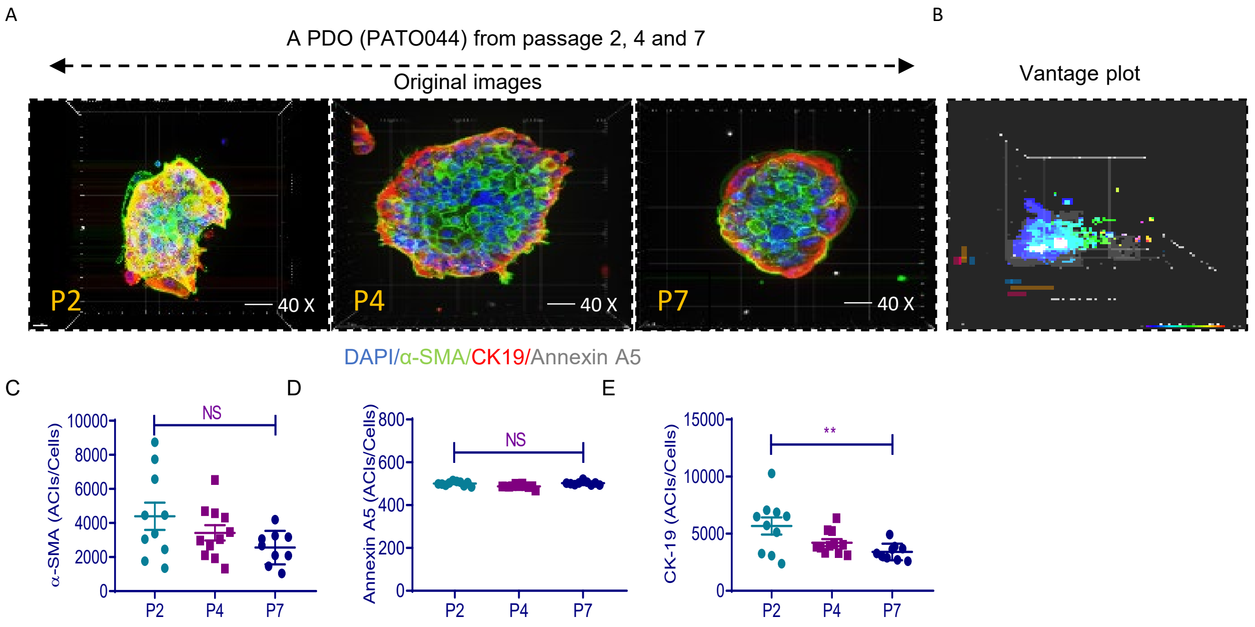

Supplementary Figure 3

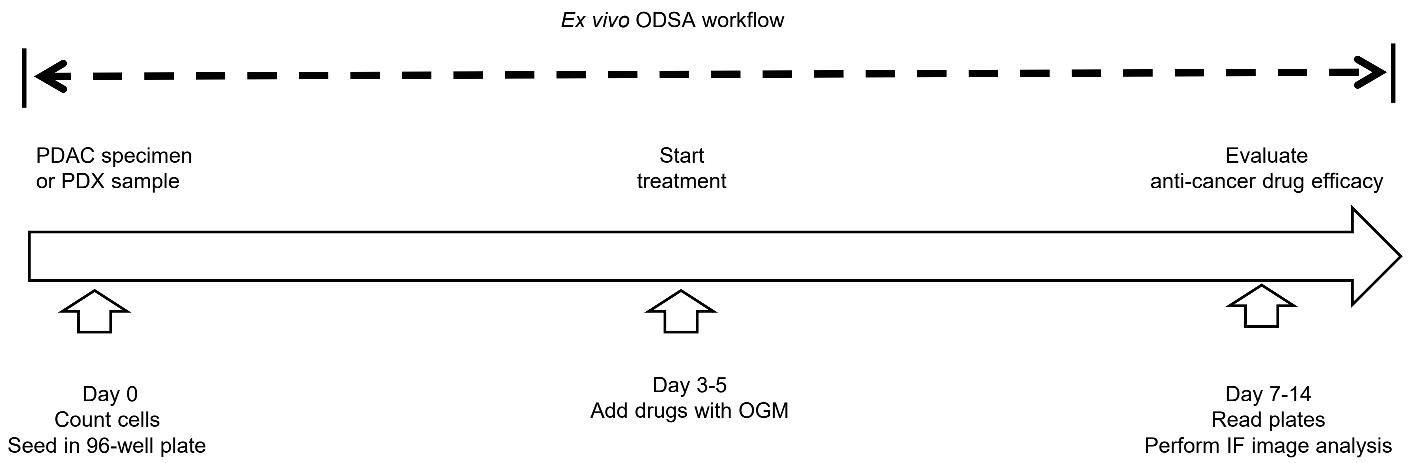

Supplementary Figure 4

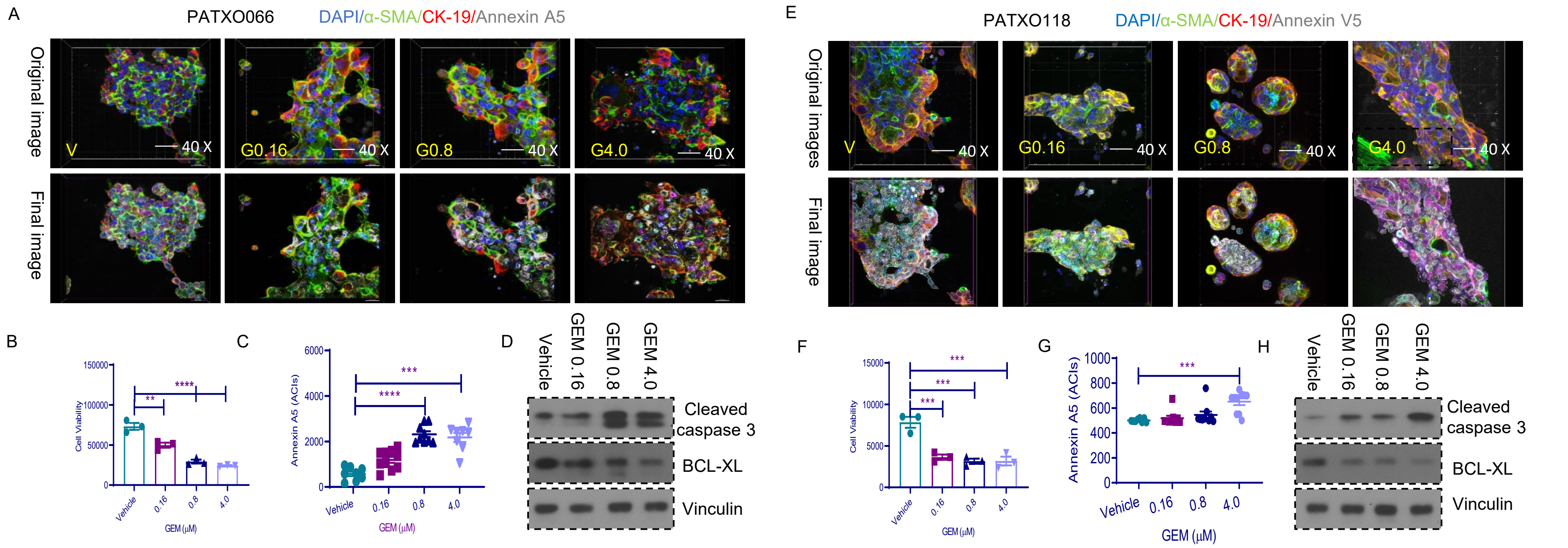

Supplementary Figure 5

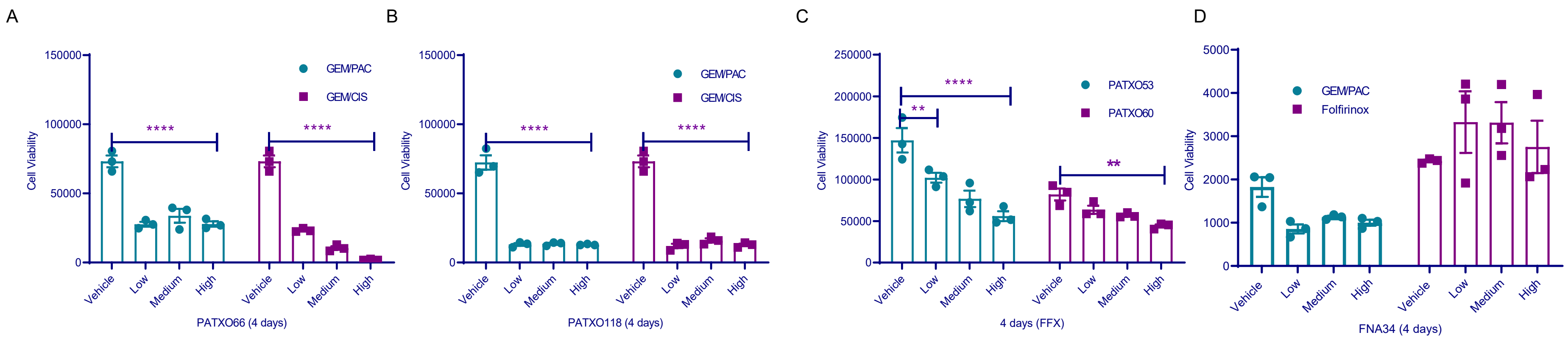

E

GEM/PAC	40%			30%			20%			10%		
	High	Medium	Low	High	Medium	Low	High	Medium	Low	High	Medium	Low
PATO032	1	1	1	1	1	1	1	1	1	1	1	1
PATO043	1	1	1	1	1	1	1	1	1	1	1	1
**PATO044**	1	1	1	1	1	1	1	1	1	1	1	1
PATO068	1	1	1	1	1	1	1	1	1	1	1	1
PATO069	1	1	1	1	1	1	1	1	1	1	1	1
**PATO073**	1	1	1	1	1	1	1	1	1	1	1	1
**PATO075**	1	1	1	1	1	1	1	1	1	1	1	1
PATO081	1	1	1	1	1	1	1	1	1	1	1	1
PATO077	0	0	0	0	0	1	1	1	1	1	1	1
PATO082	0	0	0	1	1	0	1	1	1	1	1	1
PATO072	1	1	0	1	1	0	1	1	0	1	1	0
PATO080*	1	1	0	1	1	0	1	1	0	1	1	0
FNA26	1	1	0	1	1	0	1	1	0	1	1	0
PATO084	1	0	0	1	1	0	1	1	0	1	1	1
FNA27	1	0	0	1	1	0	1	1	0	1	1	0
PATO071	1	0	0	1	0	0	1	0	0	1	0	0
PATO035	0	0	0	0	0	0	1	0	0	1	1	0
PATO062	0	0	0	0	0	0	1	0	0	1	0	0
PATO066	0	0	0	0	0	0	0	0	1	0	0	1
PATO083	0	0	0	0	0	0	1	0	0	1	1	0
PATO061	0	0	0	0	0	0	0	0	0	0	0	0

FFX	40%			30%			20%			10%		
	High	Medium	Low	High	Medium	Low	High	Medium	Low	High	Medium	Low
PATO072	1	1	1	1	1	1	1	1	1	1	1	1
PATO073	1	1	1	1	1	1	1	1	1	1	1	1
PATO077	1	1	1	1	1	1	1	1	1	1	1	1
FNA26	1	1	1	1	1	1	1	1	1	1	1	1
FNA27	1	1	1	1	1	1	1	1	1	1	1	1
**PATO069**	1	1	0	1	1	1	1	1	1	1	1	1
**PATO071**	1	1	0	1	1	1	1	1	1	1	1	1
**PATO068**	1	0	1	1	1	1	1	1	1	1	1	1
**PATO075**	1	0	1	1	1	1	1	1	1	1	1	1
PATO083	1	1	0	1	1	1	1	1	1	1	1	1
**PATO061**	1	0	0	1	0	0	1	1	1	1	1	1
PATO080*	1	0	0	1	0	0	1	0	0	1	1	1
PATO084	1	0	0	1	0	0	1	1	0	1	1	1
PATO032	0	0	0	1	0	0	1	1	0	1	1	1
**PATO082**	0	0	0	1	0	0	1	1	0	1	1	1
PATO081	0	0	0	0	0	0	1	1	1	1	1	1
**PATO043**	1	0	0	1	0	0	1	1	0	1	1	0
PATO062	0	0	0	0	0	0	0	0	0	1	1	0
PATO035	0	0	0	0	0	0	0	0	0	0	0	0
PATO044	0	0	0	0	0	0	0	0	0	0	0	0
PATO066	0	0	0	0	0	0	0	0	0	0	0	0

F

GEM/PAC	40%			30%			20%			10%		
	High	Medium	Low	High	Medium	Low	High	Medium	Low	High	Medium	Low
PATO068	1	1	1	1	1	1	1	1	1	1	1	1
PATO069	1	1	1	1	1	1	1	1	1	1	1	1
PATO071	1	0	0	1	0	0	1	0	0	1	0	0
PATO072	1	1	0	1	1	0	1	1	0	1	1	0
**PATO073**	1	1	1	1	1	1	1	1	1	1	1	1
**PATO075**	1	1	1	1	1	1	1	1	1	1	1	1
PATO080*	1	1	0	1	1	0	1	1	0	1	1	0
PATO081	1	1	1	1	1	1	1	1	1	1	1	1
PATO043	1	1	1	1	1	1	1	1	1	1	1	1
**PATO044**	1	1	1	1	1	1	1	1	1	1	1	1
PATO032	1	1	1	1	1	1	1	1	1	1	1	1
PATO084	1	0	0	1	1	0	1	1	0	1	1	1
FNA26	1	1	0	1	1	0	1	1	0	1	1	0
FNA27	1	0	0	1	1	0	1	1	0	1	1	0
PATO077	0	0	0	0	0	1	1	1	1	1	1	1
PATO082	0	0	0	1	1	0	1	1	1	1	1	1
PATO062	0	0	0	0	0	0	1	0	0	1	0	0
PATO066	0	0	0	0	0	0	0	0	1	0	0	1
PATO035	0	0	0	0	0	0	1	0	0	1	1	0
PATO083	0	0	0	0	0	0	1	0	0	1	1	0
PATO061	0	0	0	0	0	0	0	0	0	0	0	0

FFX	40%			30%			20%			10%		
	High	Medium	Low	High	Medium	Low	High	Medium	Low	High	Medium	Low
**PATO068**	1	0	1	1	1	1	1	1	1	1	1	1
**PATO069**	1	1	0	1	1	1	1	1	1	1	1	1
**PATO071**	1	1	0	1	1	1	1	1	1	1	1	1
PATO072	1	1	1	1	1	1	1	1	1	1	1	1
PATO073	1	1	1	1	1	1	1	1	1	1	1	1
**PATO075**	1	0	1	1	1	1	1	1	1	1	1	1
PATO077	1	1	1	1	1	1	1	1	1	1	1	1
PATO080*	1	0	0	1	0	0	1	0	0	1	1	1
PATO083	1	1	0	1	1	1	1	1	1	1	1	1
PATO084	1	0	0	1	0	0	1	1	0	1	1	1
FNA26	1	1	1	1	1	1	1	1	1	1	1	1
FNA27	1	1	1	1	1	1	1	1	1	1	1	1
**PATO043**	1	0	0	1	0	0	1	1	0	1	1	0
**PATO061**	1	0	0	1	0	0	1	1	1	1	1	1
PATO032	0	0	0	1	0	0	1	1	0	1	1	1
**PATO082**	0	0	0	1	0	0	1	1	0	1	1	1
PATO081	0	0	0	0	0	0	1	1	1	1	1	1
PATO062	0	0	0	0	0	0	0	0	0	1	1	0
PATO035	0	0	0	0	0	0	0	0	0	0	0	0
PATO044	0	0	0	0	0	0	0	0	0	0	0	0
PATO066	0	0	0	0	0	0	0	0	0	0	0	0

Supplementary Figure 6

A

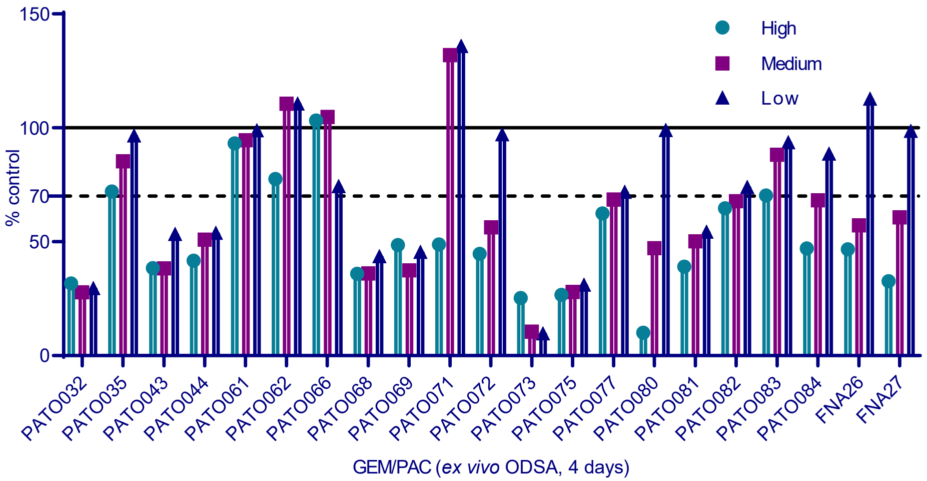

B

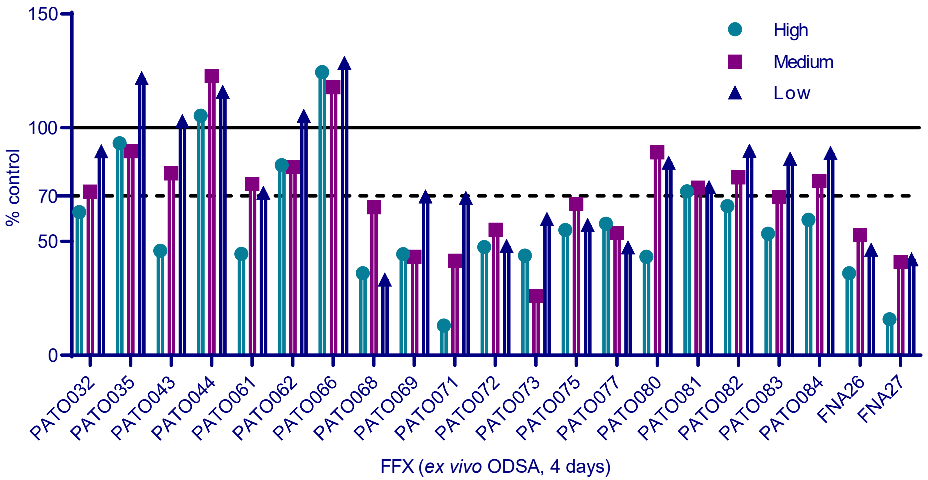

C

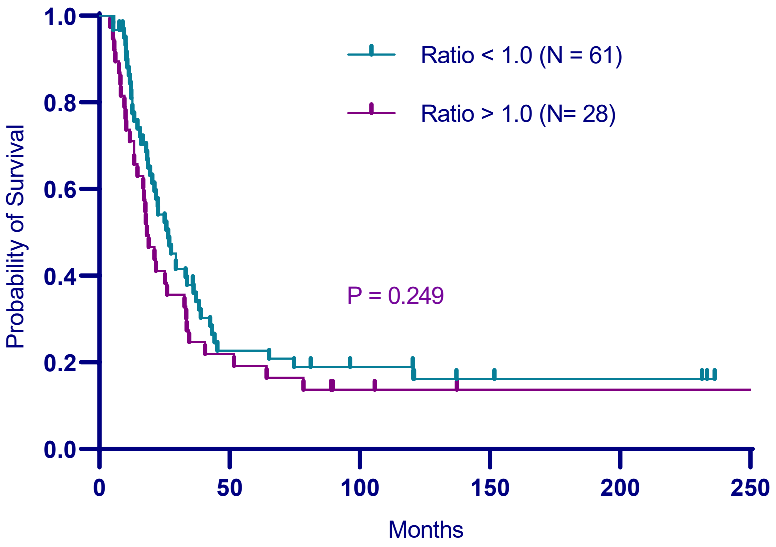

Supplementary Figure 7
